# Histopathological growth patterns and tumor-infiltrating lymphocytes in breast cancer liver metastases

**DOI:** 10.1038/s41523-023-00602-6

**Published:** 2023-12-15

**Authors:** Sophia Leduc, Maxim De Schepper, François Richard, Marion Maetens, Anirudh Pabba, Kristien Borremans, Joris Jaekers, Emily Latacz, Gitte Zels, Ali Bohlok, Karen Van Baelen, Ha Linh Nguyen, Tatjana Geukens, Luc Dirix, Denis Larsimont, Sophie Vankerckhove, Eva Santos, Rui Caetano Oliveira, Kristòf Dede, Janina Kulka, Székely Borbala, Ferenc Salamon, Lilla Madaras, A. Marcell Szasz, Valerio Lucidi, Yannick Meyer, Baki Topal, Cornelis Verhoef, Jennie Engstrand, Carlos Fernandez Moro, Marco Gerling, Imane Bachir, Elia Biganzoli, Vincent Donckier, Giuseppe Floris, Peter Vermeulen, Christine Desmedt

**Affiliations:** 1 Laboratory for Translational Breast Cancer Research, Department of Oncology, KU Leuven, Leuven, Belgium; 2 Department of Pathology, University Hospitals Leuven, Leuven, Belgium; 3 Department of Gynecological Oncology, University Hospitals Leuven, KU Leuven, Leuven, Belgium; 4 Department of Abdominal Surgery, University Hospitals Leuven, KU Leuven, Leuven, Belgium; 5 Translational Cancer Research Unit, GZA Hospitals & CORE, MIPRO, University of Antwerp, Antwerp, Belgium; 6 Department of Surgical Oncology, Institut Jules Bordet, Université Libre de Bruxelles, Brussels, Belgium; 7 Department of Anatomopathology, Institut Jules Bordet, Brussels, Belgium; 8 General Surgery Department, Centro Hospitalar e Universitario de Coimbra, Coimbra, Portugal; 9 Department of Surgical Oncology, Uzsoki Hospital, Budapest, Hungary; 10 Department of Pathology, Forensic and Insurance Medicine, Semmelweis University, Budapest, Hungary; 11 Department of Pathology, Uzsoki Hospital, Budapest, Hungary; 12 Division of Oncology, Department of Internal Medicine and Oncology, Semmelweis University, Budapest, Hungary; 13 Department of Abdominal Surgery, Erasme Hospital, Université Libre de Bruxelles, Brussels, Belgium; 14 Department of Surgical Oncology and Gastrointestinal Surgery, Erasmus MC Cancer Institute, Rotterdam, the Netherlands; 15 Division of Surgery, Department of Clinical Science, Intervention and Technology, Karolinska Institutet at Karolinska University Hospital, Stockholm, Sweden; 16 Department of Biosciences and Nutrition, Karolinska Institute, Huddinge and Karolinska University Hospital, Solna, Sweden; 17 Department of Anesthesiology, Institut Jules Bordet, Université Libre de Bruxelles, Brussels, Belgium; 18 Unit of Medical Statistics, Biometry and Epidemiology, Department of Biomedical and Clinical Sciences (DIBIC) “L. Sacco” & DSRC, LITA Vialba campus, University of Milan, Milan, Italy; 19 Department of Imaging and Pathology, Laboratory of Translational Cell & Tissue Research and University Hospitals Leuven, KU Leuven, Leuven, Belgium

**Keywords:** Breast cancer, Prognostic markers, Prognostic markers

## Abstract

Liver is the third most common organ for breast cancer (BC) metastasis. Two main histopathological growth patterns (HGP) exist in liver metastases (LM): desmoplastic and replacement. Although a reduced immunotherapy efficacy is reported in patients with LM, tumor-infiltrating lymphocytes (TIL) have not yet been investigated in BCLM. Here, we evaluate the distribution of the HGP and TIL in BCLM, and their association with clinicopathological variables and survival. We collect samples from surgically resected BCLM (*n* = 133 patients, 568 H&E sections) and post-mortem derived BCLM (*n* = 23 patients, 97 H&E sections). HGP is assessed as the proportion of tumor liver interface and categorized as pure-replacement (‘pure r-HGP’) or any-desmoplastic (‘any d-HGP’). We score the TIL according to LM-specific guidelines. Associations with progression-free (PFS) and overall survival (OS) are assessed using Cox regressions. We observe a higher prevalence of ‘any d-HGP’ (56%) in the surgical samples and a higher prevalence of ‘pure r-HGP’ (83%) in the post-mortem samples. In the surgical cohort, no evidence of the association between HGP and clinicopathological characteristics is observed except with the laterality of the primary tumor (*p* value = 0.049) and the systemic preoperative treatment before liver surgery (*p* value = .039). TIL is less prevalent in ‘pure r-HGP’ as compared to ‘any d-HGP’ (*p* value = 0.001). ‘Pure r-HGP’ predicts worse PFS (HR: 2.65; CI: (1.45–4.82); *p* value = 0.001) and OS (HR: 3.10; CI: (1.29–7.46); *p* value = 0.011) in the multivariable analyses. To conclude, we demonstrate that BCLM with a ‘pure r-HGP’ is associated with less TIL and with the worse outcome when compared with BCLM with ‘any d-HGP’. These findings suggest that HGP could be considered to refine treatment approaches.

## Introduction

Breast cancer (BC) is the most frequent cancer in women. It is estimated that 20–30% of patients with early-stage BC will develop metastases^1^. Despite advances in BC research, treatment of metastatic BC is challenging, and metastases remain the main cause of death^2^. The liver is the third most common organ for BC metastases, following bone and lung^2^, and they are reported to be present in ~50% of all patients with metastatic BC^3^. The liver is also the primary site of recurrence in 5–12% of patients with BC^3^. BC liver metastases (BCLM) are more prevalent in patients with primary triple-negative and human epidermal growth factor receptor-2 amplified (HER2^amp^) tumors^2^. Patients with liver-only or liver-dominant BC metastases can be treated with surgical resection or less-invasive local procedures such as thermal ablation, radioembolization, or surgical resection^4^.

Metastatic tumor growth in the liver relies on the interaction between cancer cells and the host microenvironment, resulting in two main histopathological growth patterns (HGP), which are assessed at the tumor-liver interface: the desmoplastic HGP (d-HGP) and the replacement HGP (r-HGP). In the d-HGP, there is a rim of desmoplastic stroma that separates the hepatocytes from the cancer cells and tumor vascularization is provided by angiogenesis. In the r-HGP, cancer cells grow into the hepatic plates and seem to replace the resident hepatocytes, thereby co-opting the sinusoidal blood vessels of the liver as a means of vascularization. Two rarer HGP, named pushing and sinusoidal, also exist in which the cancer cells literally push the hepatic plates aside, without infiltrating them and without desmoplastic reaction, or fill the sinusoidal vascular spaces, respectively^5–8^. Of note, the assessment of HGP in small biopsies is unreliable because of the frequent high level of heterogeneity of the growth pattern within the same lesion since different percentual distribution of the HGP is possible throughout the tumor-liver interface. It therefore necessitates a thorough evaluation of the whole tumor-liver interface in surgical resection specimens. The prognostic value of the HGP is well established in colorectal cancer (CRC), with d-HGP being associated with an improved progression-free (PFS) and overall survival (OS)^9^. While we recently reported a similar observation in a small series of 36 patients with surgically resected BCLM^10^, these preliminary results must be confirmed in a larger series of patients.

## Results

### Patient characteristics

Firstly, we collected 568 formalin-fixed paraffin-embedded (FFPE) samples from 125 unique metastases from 133 patients with BC who underwent surgical resection of their LM between October 2000 and September 2021 (Table 1, Supplementary Fig. 3a), further referred to as the surgical cohort. Secondly, we collected 97 FFPE samples from 84 unique LM from 23 BC patients included in two post-mortem tissue donation programs (Supplementary Fig. 3b). In the surgical and post-mortem cohorts, the median age at primary diagnosis was 47 (IQR: 18) and 50 (IQR: 20.75) years, respectively. In both cohorts, most of the primary tumors were invasive breast carcinoma of no special type (IBC-NST) (86%) and expressed ER but not HER2 (Table 1, Supplementary Table 3), with 72% (76/106) primary cancers being ER+/HER2^non-amp^, 5% (5/106) ER−/HER2^amp^, 12% (13/106) ER+/HER2^amp^ and 11% (12/106) ER−/HER2^non-amp^. In 63% (80/127) of the patients, the LM was ER+/HER2^non-amp^, in 17% (22/127) ER−/HER2^non-amp^, in 14% (18/127) ER+/HER2^amp^ and in 6% (7/127) ER-/HER2^amp^ (Table 1, Supplementary Table 3).

**Table 1:** Clinicopathological characteristics of the surgical cohort.

Clinicopathological characteristics of the primary disease						
	*n*	‘any d-HGP’	‘pure r-HGP’	OR	CI 95%	*p*
Menopausal status (post- vs pre-menopausal)				1.75	0.78–4.00	0.175
Post-menopausal	42	19 (33.3)	23 (50.0)			
Pre-menopausal	61	38 (66.7)	23 (50.0)			
Missing	30	17	13			
Age (>50 vs ≤50 years)				0.76	0.34–1.66	0.501
≤50	68	36 (60.0)	32 (69.6)			
>50	38	24 (40.0)	14 (30.4)			
Missing	27	14	13			
cT (>1 vs 1)				1.20	0.44–3.42	0.729
1	20	12 (23.1)	8 (20.0)			
2	52	27 (51.9)	25 (62.5)			
3	17	11 (21.1)	6 (15)			
4	3	2 (3.8)	1 (2.5)			
Missing	33	18	15			
cN (≥1 vs 0)				1.17	0.47–2.93	0.734
0	46	27 (51.9)	19 (47.5)			
1	37	21 (40.4)	16 (40.0)			
2	8	3 (5.8)	5 (12.5)			
3c	1	1 (1.9)	0 (0.0)			
Missing	41	22	19			
cM (1 vs 0)				1.08	0.39–2.95	0.880
0	57	35 (68.6)	22 (71.0)			
1	25	16 (31,4)	9 (29.0)			
Missing	51	23	28			
pN (1 vs 0)				2.16	0.73–6.95	0.162
0	26	16 (47.1)	10 (30.3)			
1	41	18 (52.9)	23 (69.7)			
Missing	66	40	26			
Histological subtype (ILC vs NST)				0.88	0.30–2.61	0.818
invasive ductal adenocarcinoma (NST)	106	59 (85.5)	47 (85.4)			
invasive lobular adenocarcinoma (ILC)	17	9 (13.0)	8 (14.5)			
Mucinous adenocarcinoma	1	1 (1.5)	0 (0.0)			
Missing	9	5	4			
Histological grade (2 and 3 vs 1)				0.95	0.29–3.11	0.926
1	11	6 (11.8)	5 (16.7)			
2	50	30 (58.8)	20 (66.7)			
3	16	12 (23.5)	4 (13.3)			
Missing	56	26	30			
Laterality (right vs left)				2.24	1.00–5.12	**0.049**
Bilateral	8	6 (9.4)	2 (4.3)			
Left	61	40 (62.5)	21 (45.7)			
Right	41	18 (28.1)	23 (50.0)			
Missing	23	10	13			
ER status (positive vs negative)				1.63	0.57–5.08	0.364
Negative	18	12 (18.2)	6 (12.2)			
Positive	97	54 (81.8)	43 (87.7)			
Missing	18	8	10			
HER2 status (amplified vs non-amplified)				0.76	0.25–2.16	0.607
Non-amplified	89	49 (81.7)	40 (85.1)			
Amplified	18	11 (18.3)	7 (14.9)			
Missing	26	14	12			
Neoadjuvant chemotherapy (yes vs no)				0.73	0.26–1.98	0.545
No	76	43 (69.4)	33 (80.5)			
Yes	27	19 (30.6)	8 (19.5)			
Missing	30	12	18			

Clinicopathological characteristics of liver metastasis						
ER-status (positive vs negative)				0.71	0.30–1.68	0.439
Negative	28	14 (20.0)	14 (25.0)			
Positive	98	56 (80,0)	42 (75,0)			
Missing	7	4	3			
HER2 status (amplified vs non-amplified)				0.87	0.35–2.10	0.758
Non-amplified	100	54 (77.1)	46 (80.7)			
Amplified	27	16 (22.9)	11 (19.3)			
Missing	6	4	2			
Extrahepatic metastasis (yes vs no)				2.93	0.96–9.58	0.060
No	94	58 (90.6)	36 (76.6)			
Yes	17	6 (9.4)	11 (23.4)			
Missing	22	10	12			
Time between BC diagnosis and liver surgery (continuous)				1.00	1.00–1.00	0.430
<1 month	27	18 (28.6)	9 (19.1)			
<1 year	8	7 (11.1)	1 (2.1)			
<2 years	11	7 (11.1)	4 (8.5)			
≥2 years	65	31 (49.2)	34 (70.2)			
Missing	22	11	11			
Systemic preoperative treatment before liver surgery (yes vs no)				0.41	0.17–0.96	**0.039**
No	34	12 (16.2)	22 (37.3)			
Yes	99	62 (83.8)	37 (62.7)			
First site of progression (liver vs other)				1.52	0.42–5.45	0.513
Liver (only)	86	50 (87.7)	36 (81.8)			
Liver and bone	2	0 (0.0)	2 (4.5)			
Liver, bone, and brain	1	0 (0.0)	1 (2.3)			
Other	12	7 (12.3)	5 (11.4)			
Unknown	32	17	15			

Regarding logistic regressions, the outcome is the HGP with ‘pure r-HGP’ as an event. For each variable, the reference is given on the right side of its label, and an OR > 1 indicates a positive association with ‘pure r-HGP’. *p* values  < 0.05 are considered significant and indicated in bold. *BC* breast cancer, *T* primary tumor, *N* regional lymph node, *M* distant metastasis, c clinical, *p* pathological, *CI* confidence interval, *ER* estrogen receptor, *HER2* human epidermal growth factor receptor-2, *NST* no special type, *ILC* invasive lobular carcinoma, *OR* Odds ratio.

### Distribution and heterogeneity of the histologic growth patterns

**Fig. 1:**
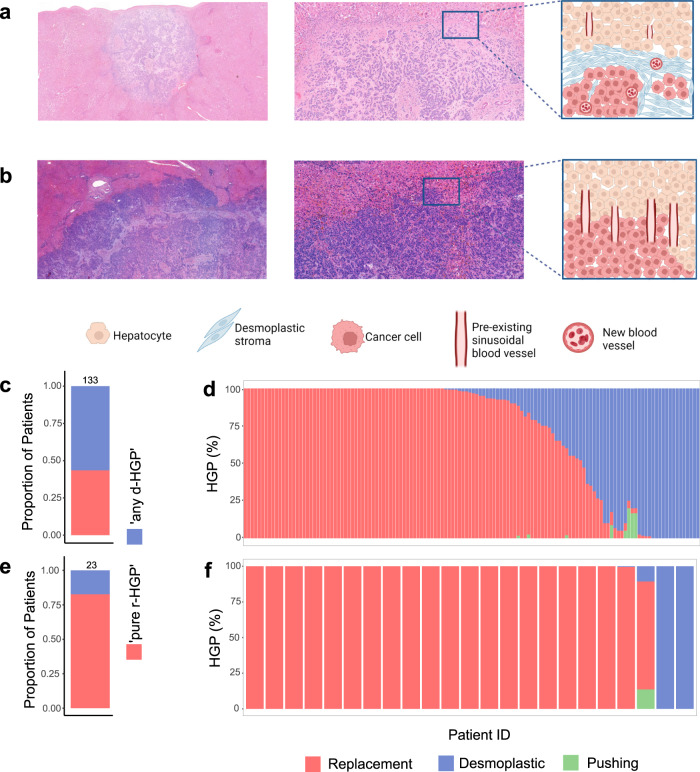
Histopathological growth pattern (HGP) in BCLM patients. **a** Hematoxylin and eosin (H&E) sections of desmoplastic liver metastasis at magnification ×2 (left), ×10 (middle) and schematic illustration (right) of the desmoplastic pattern with the desmoplastic rim between the hepatocytes and the cancer cells. **b** H&E sections of replacement liver metastasis at magnification ×2 (left), ×10 (middle) and schematic illustration of the replacement HGP (right) with direct contact between the hepatocytes and the cancer cells. **c** Proportion (%) of patients (*n* = 133) from the surgical cohort per HGP categories. ‘Any d-HGP’ (blue) and ‘pure r-HGP’ (red). **d** HGP distribution (%) in the 133 patients from the surgical cohort. **e** Proportion (%) of patients from the post-mortem cohort (*n* = 23) per HGP categories. ‘Any d-HGP’ (blue) and ‘pure r-HGP’ (red). **f** HGP distribution (%) in the 23 patients from the post-mortem cohort. Blue= desmoplastic, red= replacement, green= pushing. H&E Hematoxylin & Eosin, HGP Histopathological Growth Pattern, d Desmoplastic, r Replacement.

Figure 1a, b illustrates the two major HGP encountered in BCLM. We considered the ‘pure r-HGP’ and ‘any d-HGP’ categories, as in our previous work^10^. In the surgical cohort, we observed 59 (44%) patients with a ‘pure r-HGP’ LM and 74 (56%) patients with ‘any d-HGP’ LM (Fig. 1c, d). In the post-mortem cohort, the frequency of ’pure r-HGP’ was significantly higher as compared to the surgical cohort (*p* value < 0.001) (Fig. 1e, f), representing 19 (83%) patients. When performing exploratory analyses using the alternative cut-offs used in CRC literature^9,11–16^, we observed, in the surgical cohort, 118 (89%) patients with ‘any r-HGP’ and 15 (11%) with ‘pure d-HGP’ and 31 (26%) patients with 'dominant d-HGP' and 98 (74%) patients with 'dominant r-HGP'. In the post-mortem cohort, 21 (91%) patients with 'any-' or 'dominant-replacement' and only 2 (9%) patients with 'pure-' or 'dominant-desmoplastic' (Supplementary Fig. 5).

**Fig. 2:**
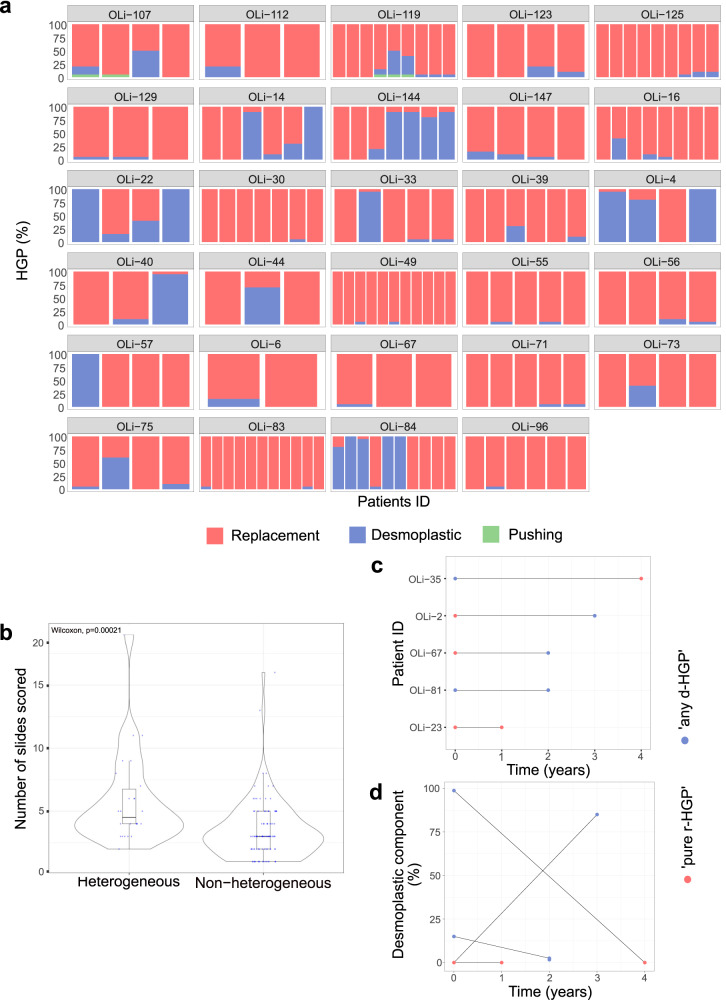
Intra-patient heterogeneity of the HGP. **a** Intra-metastasis inter-slide heterogeneity was observed in 29 (22%) patients in the surgical cohort. The proportion (%) of each HGP is given on the *y* axis. Each column represents a slide, and each box represents a patient. **b** Association between the number of slides scored (*y* axis) and the inter-slide heterogeneity (*x* axis). The significance of the association is assessed by a Wilcoxon–Mann–Whitney test between heterogeneous and non-heterogeneous (*p* value < 0.001). **c** HGP category in the five patients (OLi-35, OLi-2, OLi-67, OLi-81, OLi-23; *y* axis) who underwent two surgical resections of LM. The time (*x* axis) reflects the interval between the two liver surgeries. **d** Percentage of desmoplastic component (*y* axis) in the surgically resected LM of these 5 patients. In patients OLi-2, OLi-81, OLi-23, OLi-67, and OLi-35, HGP was assessed in 1, 2, 4, 2, and 8 slides of the first liver resection and 3, 4, 4, 3, and 1 of the second liver resection, respectively. Blue dot= ‘any d-HGP’ LM; red dot= ‘pure r-HGP’ LM. Blue= Desmoplastic; red= Replacement, green= Pushing. HGP Histopathological Growth Patterns, d-HGP desmoplastic, r-HGP replacement.

We next evaluated the intra-metastasis inter-slide heterogeneity of the HGP by comparing the different H&E slides from the same LM. Of note, we scored the HGP in more than one slide in 116 (87%) patients, and a median of three slides was evaluated for each patient (range: 2.25–5.00) and metastasis (range: 2.00–3.00), respectively, in the surgical cohort. We observed an intra-metastasis inter-slide heterogeneity in 29/133 (22%) patients (Fig. 2a), meaning that in at least 1 out of 5 patients, a single slide was not representative of the whole metastasis. Of interest, and as expected, we noticed that in the group of patients with intra-metastasis heterogeneity, the number of available slides was significantly higher than when no intra-metastasis heterogeneity was observed (*p* value < 0.001) (Fig. 2b). In the post-mortem cohort, we received one slide from 62% (8/13) of patients and two slides from 38% (5/13) of patients from the Semmelweis post-mortem program. However, in the UPTIDER cohort, we received and scored the HGP in a minimum of two slides of 90% (9/10) of patients and we analyzed a median of 8 (range: 4.75–10.75) LM per patient and 1 (range: 1.00–1.00) slide per LM. In the Semmelweis cohort, we evaluated one (range: 1.00–2.00) slide per patient and one (range: 1.00–1.00) slide per LM. We could, obviously, not detect intra-metastasis heterogeneity but found an intra-patient inter-metastasis heterogeneity in one out of the 10 UPTIDER patients (10%).

We further explored the longitudinal intra-patient heterogeneity of the HGP for five patients who underwent two surgical resections of their LM (Fig. 2c), at different timepoints. No clear evolution pattern was observed since, for one patient, the first LM was ‘any d-HGP’ and the second ‘pure r-HGP’, the reverse was seen in two patients, and the last two patients had the same HGP category in their two longitudinally resected LM. Also, we did not record a clear-cut decrease/increase in desmoplastic components depending on time (Fig. 2d).

### Association between histopathological growth patterns and standard clinicopathological characteristics

To evaluate whether the standard clinicopathological variables were associated with the HGP in the surgical cohort, we first assessed the associations using a logistic regression adjusted by center (Table 1). We observed an association between the ‘any d-HGP’ and the left laterality of the primary tumor (OR: 2.24 (1.00–5.12); *p* value = 0.049) and the administration of a systemic pre-operative treatment of the LM, which was given to 99/133 (74%) of the patients (OR: 0.41 (0.17–0.96); *p* value = 0.039). Of note, the type of treatment was missing for 20 patients from Centro Hospitalar e Universitario de Coimbra and 12 patients from Erasmus MC. Chemotherapy was the most commonly administered systemic treatment in 54/80 (67.5%) patients. Other treatments are listed in Supplementary Table 4. Sixty-two percent of the 34 patients who did not receive a systemic pre-operative treatment had LM with ‘pure r-HGP’. These 34 patients were generally young at primary diagnosis (52% were younger than 50 years old) and had a primary BC and LM, which were ER+/HER2^non-amp^ in 87.5% (21/4) and 67% (22/33) of the patients.

We further observed a trend towards an association between ‘pure r-HGP’ and the presence of extrahepatic metastasi(e)s (OR: 2.93 (0.96–9.58); *p* value = 0.060). Of note, we did not observe any association either with the ER, HER2, or the histological subtype status of the primary tumor or LM.

When repeating the analyses using the two other exploratory cut-offs, no association was seen when considering the ‘any r-HGP’ vs ‘pure d-HGP’ categories (Supplementary Table 1). When considering the ‘dominant r-HGP’ vs ‘dominant d-HGP’, we only observed an association between the ‘dominant r-HGP’ and the time elapsed between BC diagnosis and LM surgery (OR: 1.00 (1.00–1.00); *p* value = .023) and the lymph nodes involvement (OR: 4.22 (1.32–14.60), *p* value = 0.015) (Supplementary Table 2). We also confirmed the ‘dominant d-HGP’ was associated with the systemic preoperative treatment before liver surgery (OR: 0.29 (0.08–0.87); *p* value = 0.026).

### Evaluation of tumor-infiltrating lymphocytes in BC liver metastases

Here, we aimed to assess the proportion of TIL in BCLM and investigate whether there are differences according to the HGP categories. We first used the well-established guidelines^17^ for scoring TIL in BCLM. However, using this method, we observed a poor inter-pathologist concordance between the three pathologists (P) (P2 vs P1 concordance correlation coefficient, CCC = 0.38; P2 vs P3 CCC = 0.41; P1 vs P3 CCC = 0.38) on 20 randomly chosen samples, with 5 scoring fields per metastasis (Supplementary Fig. 6a). This was probably due to differences in the interpretation of current TIL guidelines^17^ applied in the liver. For this reason, we refined the method for scoring the TIL taking into consideration the HGP (Supplementary Methods). Using the refined method, we obtained a very good CCC between the scores of the three pathologists on the same 20 randomly chosen samples (P3 vs P1 CCC = 0.98; P3 vs P2 CCC = 0.97; P1 vs P2 CCC = 0.99) as well as on the first 213 samples scored with this new method (P3 vs P1 CCC = 0.98; P3 vs P2 CCC = 0.97; P1 vs P2 CCC = 0.99) (Supplementary Fig. 6b, c).

**Fig. 3:**
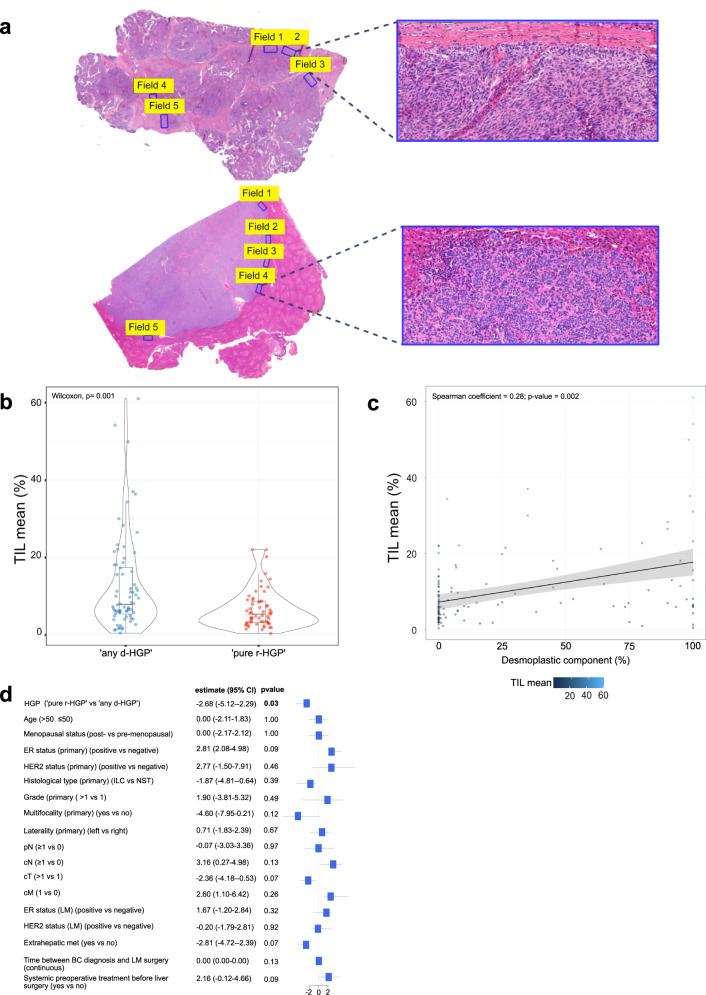
TIL in LM from BC patients in the surgical cohort. **a** H&E section from LM at magnification ×0.5 with the five representative fields scored, and a ×20 magnification representation of one field scored for the desmoplastic HGP (top) and replacement HGP (bottom). Blue frame delineates the area to score and is defined by the outer cancer cells, including the desmoplastic rim, when present. **b** TIL mean distribution (%; *y* axis) according to ‘any d-HGP’ *vs* ‘pure r-HGP’. Lower TIL infiltration in ‘pure r-HGP’ in comparison with d-HGP (*p* value = 0.001). Significance of the association is assessed by a Wilcoxon–Mann–Whitney test. **c** Continuous distribution of the TIL (%; *y* axis) according to the percentage of desmoplastic growth pattern (Spearman correlation coefficient= 0.28; *p* value = 0.002). **d** Association between the TIL and the clinicopathological variables adjusted by center. Only ‘pure r-HGP’ was associated with lower immune infiltrates (*p* value = .03). For each variable, the reference is given on the right side, and a positive estimate indicates a lower TIL level in the reference. The significance of the association is assessed by quantile regression. Only 95% CI are clipped at –5 and 5. Blue= ‘any d-HGP’; red= ‘pure r-HGP’. HGP histopathological growth pattern, TIL tumor infiltrating lymphocytes, BC breast cancer, T primary tumor, N regional lymph node, M distant metastasis, c clinical, p pathological, ER estrogen receptor, HER2 human epidermal growth factor receptor-2, NST no special type, ILC invasive lobular carcinoma.

In the surgical cohort and across all LM, a median of 6.60% (range: 0.40–61.00%) of TIL was observed. We investigated whether TIL levels differed according to the HGP categories. We observed lower TIL levels in LM with ‘pure r-HGP’ as compared to LM with ‘any d-HGP’ (*p* value = 0.001) (Fig. 3b). A Spearman correlation coefficient of 0.28 (*p* value = 0.002) was observed between continuous TIL levels and the percentage of desmoplastic HGP. As for the HGP, we also evaluated the intra-metastasis inter-slide heterogeneity in TIL. Heterogeneity, defined here arbitrarily as a difference of at least 5 or 10% between two slides, was observed in 76 (57%) patients and 36 (27%) patients, respectively (Supplementary Fig. 8).

In the post-mortem cohort, we observed a median of 2.50% (range: 0.00–9.80%), which is significantly lower than what we observed in the surgical cohort (*p* value < 0.001) (Supplementary Fig. 9). In the post-mortem cohort, intra-patient inter-metastasis heterogeneity was observed in 4 (28.9%) patients and one (7%) patient using the 5% and 10% criterion, respectively (Supplementary Fig. 10).

In the surgical cohort, continuous TIL levels were found to be lower in LM with ‘pure r-HGP’ (*p* value = 0.03) (Fig. 3d). We further illustrated the immune infiltrates using IHC with antibodies against CD3, CD8 and CD20, showing exemplary cases of LM with ‘any d-HGP’ with elevated immune infiltrates concentrated in the desmoplastic rim and of LM with ‘pure r-HGP’ with only few immune cells present (Supplementary Fig. 11).

### Survival analyses

**Fig. 4:**
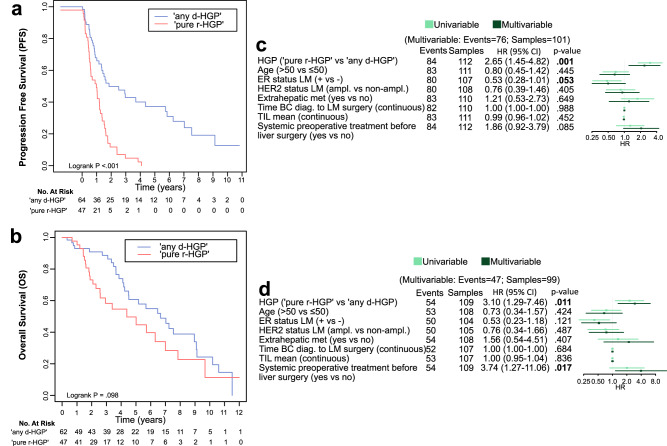
Survival analyses. **a**, **b** Survival curves (Kaplan–Meier) using the ‘any d-HGP’ versus ‘pure r-HGP’ categories. The horizontal axis (*x* axis) represents time in years, and the vertical axis (*y* axis) shows the probability of surviving. **a** Association between Progression-Free Survival (PFS) and the HGP categories (Logrank *p* < 0.001). Start event is the first liver resection; end event is the progression or death. **b** Association between Overall Survival (OS) and the HGP categories (Logrank *p* = 0.098). Start event is the first liver resection; end event is the death or last follow-up. **c** Univariable (light green) and multivariable (dark green) Cox regression analyses for PFS. ‘Pure r-HGP’ (HR: 2.65 (1.45–4.82); *p* value = .001) was significantly associated with a worse PFS. **d** Univariable (light green) and multivariable (dark green) Cox regression analyses for OS. ‘Pure r-HGP’ (HR: 3.10 (1.29–7.46); *p* value = .011) and systemic preoperative treatment before liver surgery (HR: 3.74 (1.27–11.06); *p* value = .017) were significantly associated with worse OS. The multivariable model included: HGP (‘pure r-HGP’ vs ‘any d-HGP), age at primary diagnosis (>50 vs ≤50), ER status of the LM (+ vs −), HER2 status of the LM (ampl. vs non-ampl.), presence of extrahepatic metastasis (yes vs no), time between BC diagnosis (continuous, per one-year increase), LM surgery and TIL (continuous, per one percent increase) and systemic preoperative treatment before liver surgery (yes vs no). Blue= Desmoplastic; red= Replacement; light green= univariable; dark green= multivariable. HR hazard ratio, CI confidence interval, HGP histopathological growth pattern, d-HGP desmoplastic, r-HGP Replacement, met. metastasis, BC breast cancer, LM liver metastasis, TIL tumor infiltrating lymphocytes, diag. Diagnosis, ampl. Amplified, non-ampl. Non-amplified, PFS progression-free survival, OS overall survival.

We performed survival analyses to investigate the association between the HGP categories, TIL, and survival in the surgical cohort, considering PFS and OS as survival endpoints. The Kaplan–Meier curves are illustrated in Fig. 4a, b. In the multivariable Cox regression analysis for PFS (Fig. 4c), only the HGP survival was significantly associated with PFS (HR: 2.65; CI: (1.45–4.82); *p* value = 0.001), with the ‘pure r-HGP’ being associated with worse survival. However, a trend towards an association was also observed between the ER status of the LM (HR: 0.53; CI: (0.28–1.01); *p* value = 0.053) and the PFS. In the multivariable analyses for OS (Fig. 4d), ‘pure r-HGP’ was also associated with worse survival (HR: 3.10; CI: (1.29–7.46), *p* value = 0.011). Here, the systemic preoperative treatment was also significantly associated with worse survival (HR: 3.74; CI: (1.27–11.06); *p* value = 0.017). The HER2 status of the LM was not significantly associated with the PFS and OS at the multivariable level. We further conducted exploratory survival analyses using the two other cut-offs, which are reported in Supplementary Fig. 12.

## Discussion

In this study, we assembled, to the best of our knowledge, the largest series of BCLM from patients who either underwent surgical resection of their metastases (surgical cohort) or who participated in post-mortem tissue donation programs (post-mortem cohort). This allowed us to evaluate resection specimens to assess HGP and TIL.

Firstly, we assessed HGP as a relative proportion of the interface for each growth pattern present in the metastasis as it has been defined in international guidelines^16,18^. We then categorized the HGP into ‘pure r-HGP’ (i.e., 100% of the tumor-liver interface is replacement) and ‘any d-HGP’ (i.e., at least 1% of the tumor-liver interface is desmoplastic), in line with our previous study^10^. In the surgical cohort, we observed LM with ‘any d-HGP’ in 56% of the patients and ‘pure r-HGP’ in 44%. These percentages are in line with what we observed before in our smaller series of 36 patients^10^. We further observed an intra-metastasis inter-slide heterogeneity in ~1 out of 5 patients. These results demonstrate the necessity to evaluate the tumor-liver interface as extensively as possible, while the biological or clinical relevance of this heterogeneity is unclear at this stage.

So far, it is unknown how these growth patterns evolve over time and under the pressure of treatment. One hypothesis is that r-HGP is the default growth pattern, when cancer cells can outcompete the hepatocytes^16^. In a later stage, treatment or other external factors could weaken the cancer cells followed by a switch from r-HGP to d-HGP with a desmoplastic rim as an injury reaction of the liver^19–21^. Some studies in CRC indeed suggest that chemotherapy can lead to desmoplastic growth in patients with replacement LM^20,21^ and this is confirmed by our results here with an interesting association between the systemic preoperative treatment before the liver surgery and the ‘any d-HGP’. No evident association was however observed between the growth patterns and ER or HER2 status of the primary tumor or of the LM, providing no support to the hypothesis of an influence of the biology related to ER and HER2-specific pathways. The lack of an association between the HGP and hormone receptor status is remarkable, given the overall impact of ER and HER2 on tumor biology and clinical course. In the post-mortem cohort, we observed a higher prevalence of ‘pure r-HGP’ as compared with the surgical cohort, suggestive of a more aggressive disease at the end of life in these patients.

So far, HGP has mainly been evaluated in surgically resected LM from patients with CRC^16^. In these patients, a higher prevalence of LM with pure desmoplastic HGP is observed, with 21% of ‘pure d-HGP’ (i.e. 100% of the tumor-liver interface is replacement) being reported or 53% with the so-called ‘predominant desmoplastic’ (i.e. > 50% of the tumor-liver interface is desmoplastic). We therefore also explored these HGP and observed 11% LM with ‘pure d-HGP’ and 26% with dominant-desmoplastic. This suggests that the cancer type (CRC vs BC), the selection criteria for liver surgery, and/or the type of systemic treatment may have an influence on the HGP of the LM.

Secondly, we evaluated the TIL on H&E slides as a surrogate of the immune infiltrates in the BCLM. To this end, we refined the existing scoring guidelines^17^ to acknowledge the morphological differences between the desmoplastic and the replacement HGP and to ensure reproducibility across pathologists. We consistently observed a lower proportion of TIL in ‘pure r-HGP’ as compared to in ‘any d-HGP’ in both cohorts. We, however observed even lower TIL levels in the LM with ‘pure r-HGP’ in the post-mortem cohort when compared with the surgical cohort. In the LM with a desmoplastic HGP, TIL was mainly located in the fibrous rim. In the surgical cohort, higher TIL levels were associated with ‘any d-HGP’. Also, we observed some intra-metastasis inter-slide heterogeneity regarding TIL. In the surgical cohort, we observed in at least one third of the patients TIL heterogeneity when considering a difference of 10% between different slides of the same LM. These results, the localization of the TIL and the inter-slide variability, suggest that evaluating TIL in a core biopsy of a LM might not give a reliable assessment of the TIL present across the whole metastasis. This was less evident in the post-mortem cohort in which the immune infiltration was generally low and differences above 10% rare.

Thirdly, we showed an association between ‘pure r-HGP’ and worse PFS and OS at the multivariable level. To be able to use this marker in the clinic and potentially use it to select patients who could benefit from surgical resection of their LM, the HGP of the LM should be reliably assessed before liver surgery. In the past few years, two teams have focused their attention on the identification of the HGP of LM of CRC^22–24^ using CT and MR imaging. However, more studies in this field are needed and we are starting a dedicated study on BCLM soon. In our study, we observed an association between TIL and HGP, but there was no association between TIL levels and survival.

Our study has several limitations, mostly inherent to its retrospective nature and the long time span covered, including the lack of clinical data in some patients. One additional limitation is that, given the criteria applied to select the patients who will undergo surgical resection of their LM, we might not have a cohort that is representative of all patients with BCLM. This would explain why we do not observe an increase in patients with HER2^amp^ or TNBC disease in our surgical cohort as expected based on the literature^2^. Additionally, only FFPE samples were available from the surgically resected LM, precluding additional advanced molecular analyses. To tackle this limitation, we are currently starting a prospective multi-centric study (NCT05720676) that will allow us to collect well-annotated mirrored fresh, fresh frozen, and formalin-fixed samples of the center of the LM, the tumor-liver interface, and the adjacent normal liver parenchyma in order to provide a comprehensive characterization of these metastases and their microenvironment using multiple single-cell and spatial omics technologies.

To conclude, our study represents the largest study evaluating HGP and TIL in BCLM. Approximately half of the surgically resected LM show ‘any d-HGP’, which is associated with more TIL and pre-operative systemic treatment as well as a better prognosis. The higher prevalence of ‘pure r-HGP’ in the post-mortem cohort also suggests that a more advanced stage of the disease is associated with an increase in ‘pure r-HGP’, reflecting a more immunosuppressed microenvironment. However, further studies are needed to biologically characterize the different HGP, to identify them pre-operatively, and to validate their clinical relevance.

## Methods

### Patients and LM samples

The study currently includes clinical data and samples from patients who had a diagnosis of BCLM from (1) a retrospective cohort of 133 patients who underwent surgical resection of their LM in one of the eight participating hospitals (University Hospitals Leuven—Leuven, Belgium; Institut Jules Bordet - Brussels, Belgium; Erasme Hospital - Brussels, Belgium; GZA Ziekenhuizen - Antwerp, Belgium; Centro Hospitalar e Universitario de Coimbra - Coimbra, Portugal; Uzsoki Hospital - Budapest, Hungary; Erasmus MC Cancer Institute - Rotterdam, The Netherlands; Karolinska Institutet - Stockholm, Sweden) between October 2000 and September 2021 (Supplementary Fig. 3a), and, (2) a post-mortem cohort of 23 patients from two autopsy programs currently ongoing (UPTIDER, KU/UZ Leuven—Leuven, Belgium, NCT04531696; and Semmelweis University - Budapest, Hungary) (Supplementary Fig. 3b). Both parts of this study received central ethical approval (S64812; 25/03/21 and S64813;28/08/2022), respectively, Ethics Committee (EC) from UZ Leuven, Belgium). Local EC approvals were further obtained, and material and data transfer agreements were put in place. No informed consent form (ICF) was requested for the retrospective surgical cohort, as a waiver was granted by the respective institutional ethics committees, given that many patients already passed away or further progressed. All patients from the UPTIDER cohort signed an ICF. The study was performed in accordance with the Declaration of Helsinki.

We considered all the H&E sections available for each BCLM, totaling 568 H&E sections for the 133 patients from the surgical cohort and 97 for the post-mortem cohort. In the surgical cohort, we evaluated one LM for 125/133 patients, three patients had 2 LM, which were surgically resected at the same time, and five patients had two different surgical resections. In UPTIDER, the median number of slides we evaluated per patient was 8 (range: 4.75–10.75) but only one (range: 1.00–1.00) slide per unique metastasis, while only one (range: 1.00–2.00) slide per patient and unique metastasis was available for the patients from the Semmelweis post-mortem program.

A homogeneous set of clinicopathological data has been retrieved from local medical files. These data include but are not limited to the age of the patient and menopausal status at primary diagnosis, BC histopathological parameters of the primary tumor (estrogen receptor (ER) status, HER2 status, histological type, histological grade, laterality, and multifocality), characteristics of the LM (hormone receptor status, HER2 status), the presence of extrahepatic metastasis, treatment, and outcome. Most of the clinicopathological data were missing for the 20 patients from Portugal since these patients were treated and followed up after surgery in other hospitals than where the resection of the LM took place.

### Scoring of the histopathological growth patterns

The HGP of LM was centrally scored by an experienced pathologist (P.V.) blinded to the clinicopathological and outcome data according to the international guidelines^18^. In short, the metastatic tumor-liver interface was identified on standard H&E sections at low power magnification, avoiding large portal tracts, zones of preexisting scarring, or subcapsular location. The whole interface was then assessed at a higher power assigning to each HGP a relative proportion (expressed as a percentage) of the whole perimeter of the metastatic tumor. In the case of large metastases, the HGP scoring was repeated for each single section, and the average HGP score was derived for each patient. Lastly, patients were stratified into two groups: ‘pure r-HGP’ and ‘any d-HGP’. The other two rarer HGP (pushing and sinusoidal) were documented but did not influence the acknowledged definitions reported in our previous work^10^. For exploratory purposes, we also considered alternative cut-offs and definitions previously used for patients with CRC^16^: (i) ‘pure d-HGP’ (i.e., 100% of the tumor-liver interface is desmoplastic) versus ‘any r-HGP’ (i.e., at least 1% of the tumor-liver interface is replacement), and (ii) 'dominant-replacement' (i.e., 51% of the tumor-liver interface is replacement) versus 'dominant-desmoplastic' (i.e., 51% of the tumor-liver interface is desmoplastic).

### Scoring of the tumor-infiltrating lymphocytes

TIL was first scored by three experienced pathologists (P.V., G.F., and M.D.S.) using the current reported guidelines for scoring TIL in BC metastases^17^, given the lack of inter-pathologist (P) concordance (see Results Section) using the CCC, we defined a new scoring method (Supplementary Method) for scoring the TIL in BCLM introducing a modification of the existing guidelines. This modification allows TIL scoring in BCLM both on glass slides as well as on digitalized whole slides images of H&E sections. Five representative fields with vital carcinoma and adjacent liver parenchyma were evaluated for each slide, from which the average TIL score was derived. For each field, the respective growth pattern was noted. The scoring was performed at the interface between metastatic BC and liver (i.e., outer margin) using a ×20 objective on standard microscopy, or a digital field with the major side of 800–1000 µm in length. For r-HGP, the scoring area was obtained by defining an imaginary line joining the two most outer cancer cells at the invasive front that touched (or crossed) the upper margin of the field of view (Supplementary Fig. 1b). Thus, all TIL present in the tissue below this line were included in the scoring. For the d-HGP, the scoring area was found below the imaginary tangential line passing through the outermost point of the desmoplastic rim (which, importantly, included lymphocytic infiltration, when present) (Supplementary Fig. 1a). Small portal tracts falling below the imaginary outer margin of the BCLM were included, but preexisting fibrous structures/capsules were excluded, as well as large blood vessels and necrotic areas. The TIL score (%) indicates the relative surface area of the non-epithelial component of the metastasis covered by lymphocytes (Supplementary Fig. 2).

### Statistical analyses

Associations between HGP and clinicopathological characteristics were assessed using the logistic regression (adjusted by center, Supplementary Fig. 4). Evaluation of the inter-observer variability for TIL scores was assessed using the Bland-Altman method, Passing-Bablok regression analyses, CCC (continuous variables). The association between TIL and clinicopathological characteristics was assessed using quantile regression (adjusted by the center). The associations between HGP, PFS, and OS were visualized using Kaplan–Meier curves and further assessed using univariable and multivariable Cox proportional hazard regressions. PFS was defined as the time from first liver resection to the time of progression or death, and OS was defined as the time from first liver resection to the time of death of any cause. The multivariable model included: HGP (‘pure r-HGP’ vs ‘any d-HGP), age at primary diagnosis (>50 vs ≤50), presence of extrahepatic metastasis (yes vs no), time between BC diagnosis (continuous, per one-year increase) and LM surgery, ER (+ vs −), HER2 (amplified vs non-amplified) and TIL status (continuous, per one percent increase) of the LM. For both PFS and OS endpoints, we used the date of the (first) liver resection as the start date. All analyses were stratified when possible or otherwise adjusted for the center. We used the R version 4.2.2.

### Reporting summary

Further information on research design is available in the Nature Research Reporting Summary linked to this article.

### Supplementary information

Supplementary Material

Reporting Summary

## Data Availability

The data that support the findings of this study are available upon request to the corresponding author after the signature of a Data Access Agreement. Due to the personal nature of the information contained, only the non-sensitive data are publicly available, and the sensitive data has been modified before being published on the Code Ocean capsule.

## Code availability

The code, together with modified data, is available online on code ocean (https://codeocean.com/capsule/5345389/tree/v1, 10.24433/CO.6616975.v1).
