# Supplementary material for: Histopathological growth patterns and tumor-infiltrating lymphocytes in breast cancer liver metastases
Source: NPJ Breast Cancer. 2023 Dec 15;9:100. doi: 10.1038/s41523-023-00602-6 (PMC10724185; doi:10.1038/s41523-023-00602-6)
Supplement: Supplementary file 1 — Supplementary Material [file 41523_2023_602_MOESM1_ESM.pdf]

Supplementary information to "*Histopathological growth patterns and tumor infiltrating lymphocytes in breast cancer liver metastases*" by Leduc S., De Schepper M. and Richard F. *et al.*

# Table of Contents


**Supplementary Method**: The evaluation of tumor infiltrating lymphocytes (TIL) in breast cancer liver metastasis: proposal to adapt the current guidelines to the histopathological growth patterns. ........2

Introduction........2

Methods ........2

**Supplementary Figures**........ ..3

**Supplementary Figure 1** ........ 3

**Supplementary Figure 2** ........ 6

**Supplementary Figure 3** ........ 7

**Supplementary Figure 4** ........ 8

**Supplementary Figure 5** ........ 9

**Supplementary Figure 6** ........10

**Supplementary Figure 7** ........11

**Supplementary Figure 8** ........11

**Supplementary Figure 9** ........12

**Supplementary Figure 10** ........12

**Supplementary Figure 11** ........13

**Supplementary Figure 12** ........14

**Supplementary Tables**........ .16

**Supplementary Table 1** ........16

**Supplementary Table 2** ........18

**Supplementary Table 3** ........20

**Supplementary Table 4** ........21

**Supplementary Table 5** ........22

**Supplementary Method**

**The evaluation of tumor infiltrating lymphocytes (TIL) in breast cancer liver metastasis: proposal to adapt the current guidelines to the histopathological growth patterns.**

## Introduction

Guidelines to ensure the reproducibility and reliability of the assessment of the tumor infiltrating lymphocytes (TIL) in breast cancer (BC) and metastasis have been published by Salgado *et al.* in 2014(1). However, when using these guidelines to score the TIL in breast cancer liver metastasis (BCLM), we faced some difficulties to reach a good concordance between three experienced pathologists (P.V., G.F. and M.D.S.). Indeed, BCLM present with two main histopathological growth patterns (HGP): the replacement (r-HGP) and the desmoplastic (d-HGP) growth pattern. These HGPs have a different morphology, one of the differences being a distinct tissue location of the immune infiltrate, for example in LM from CRC(2). While in the replacement HGP, the immune cells are located at the interface between the cancer cells and the hepatocytes, in the desmoplastic growth pattern, the fibrous rim that separates cancer cells from hepatocytes contains most of the immune cells. Importantly, these HGP-related differences are not acknowledged in the original guidelines by Salgado *et al.*[1], which may also explain the high degree of inter-observer discordance we experienced when using these guidelines for scoring the TIL in BCLM.

In this study, we aim at refining the guidelines to score the TIL in BCLM acknowledging the morphological differences, including the location of the immune infiltrate, between both HGP.

## Methods

*Scoring of the TIL*

The first step is to identify the HGP present in the BCLM (Figure 1). In the second step five representative regions of the HGP present at the invasive front are selected for the TIL scoring. The third step is a key step since the scoring area will be defined depending on the HGP present. In case of d-HGP (Figure 1A), an imaginary line passing through the most outer point of the desmoplastic rim is drawn. Importantly this imaginary line in the d-HGP should include the inflammatory infiltrate, if present. In case of r-HGP (Figure 1B-, a line passing through the two most outer cancer cells of the field is drawn. The imaginary lines in both HGP should overlap or cross with the upper border of the microscopic field using a 20x magnification, all the tissue present and below the imaginary line is then scored for the TILs. In case of pushing growth pattern, the method of r-HGP is applied. The final score

per slide is calculated by the average of the five fields. In case of multiple slides for the same metastasis, we have also calculated the average of the scores of each slide. Portal tracts falling under the above-mentioned lines were included in the scoring.

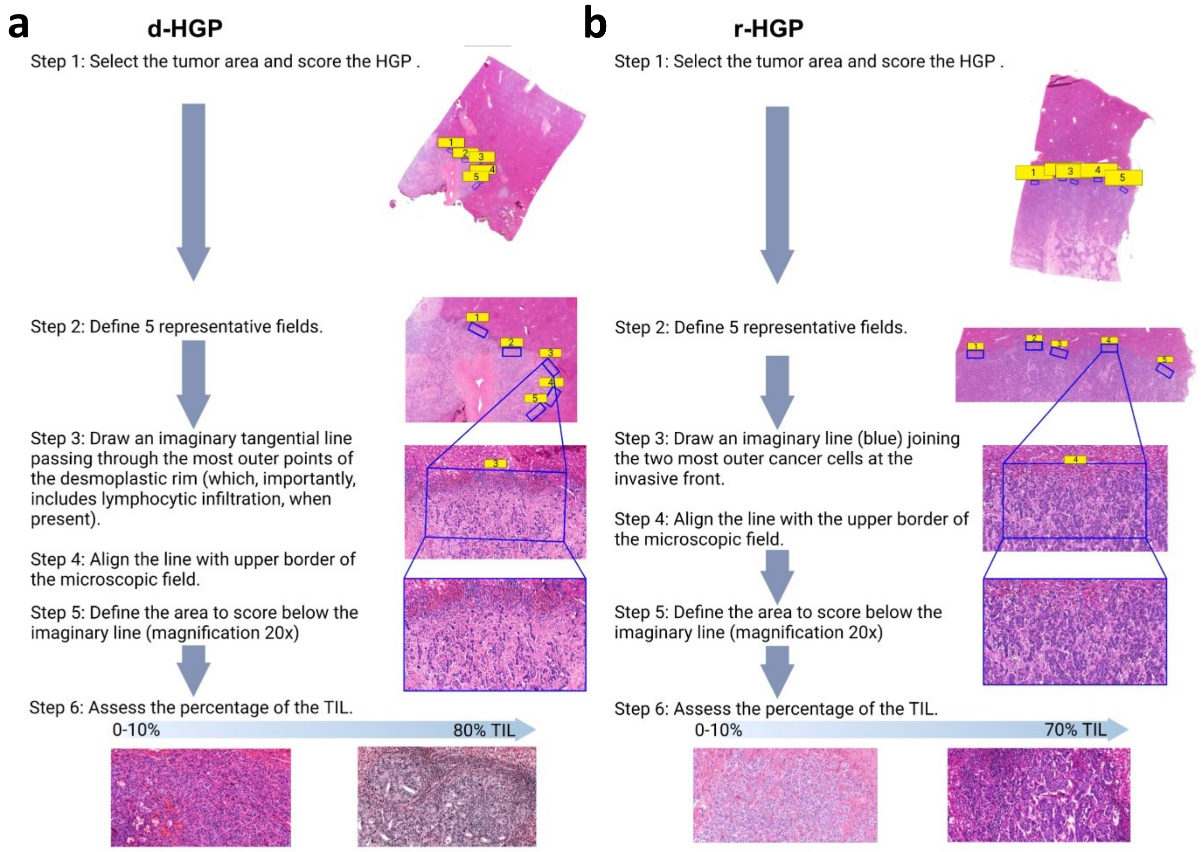


**Supplementary Figure 1**: Method to score the TIL in BCLM. **a.** In BCLM with d-HGP, we select the tumor area, score the HGP (step 1) and define five representative fields (step 2). We draw an imaginary tangential line (blue) passing through the most outer point of the desmoplastic rim which, importantly, included lymphocytic infiltration, when present (step 3) and align this line with the upper border of the microscopic field (step 4). Once the representative fields are defined below the imaginary lines (blue) at a magnification 20x (step 5), we assess the percentage of TIL (step 6). **b.** In BCLM with r-HGP, we select the tumor area, score the HGP (step 1) and define five representative fields (step 2). We draw an imaginary line (blue) passing through the most outer cancer cells (step 3) and align this line with the upper border of the microscopic field (step 4). Once the representative fields are defined below the imaginary lines (blue) at a magnification 20x (step 5), we assess the percentage of TIL (step 6). Abbreviations: TIL= Tumor Infiltrating Lymphocytes; BCLM= Breast Cancer Liver Metastasis; d-HGP= Desmoplastic; r-HGP= Replacement.

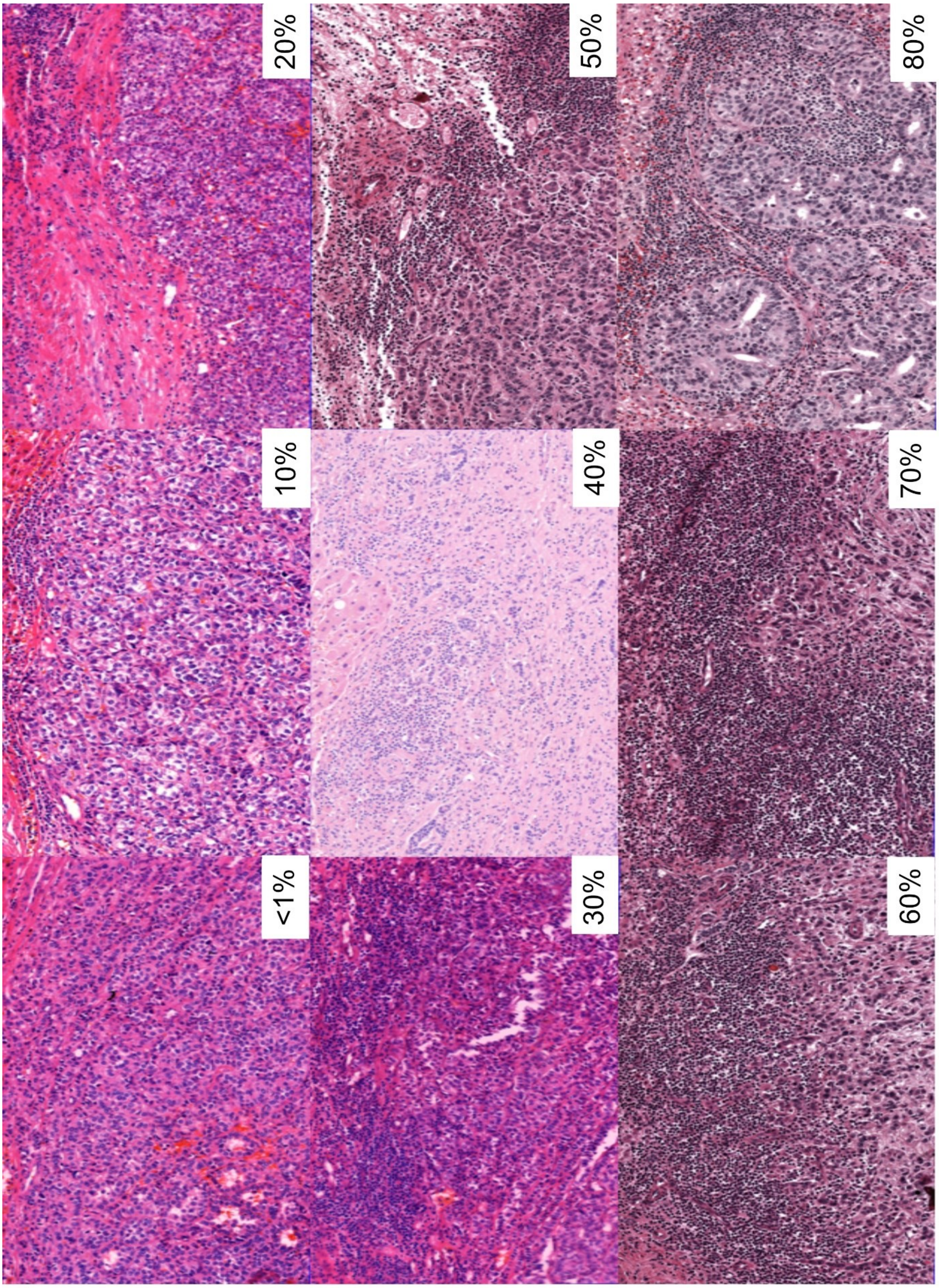


**a**

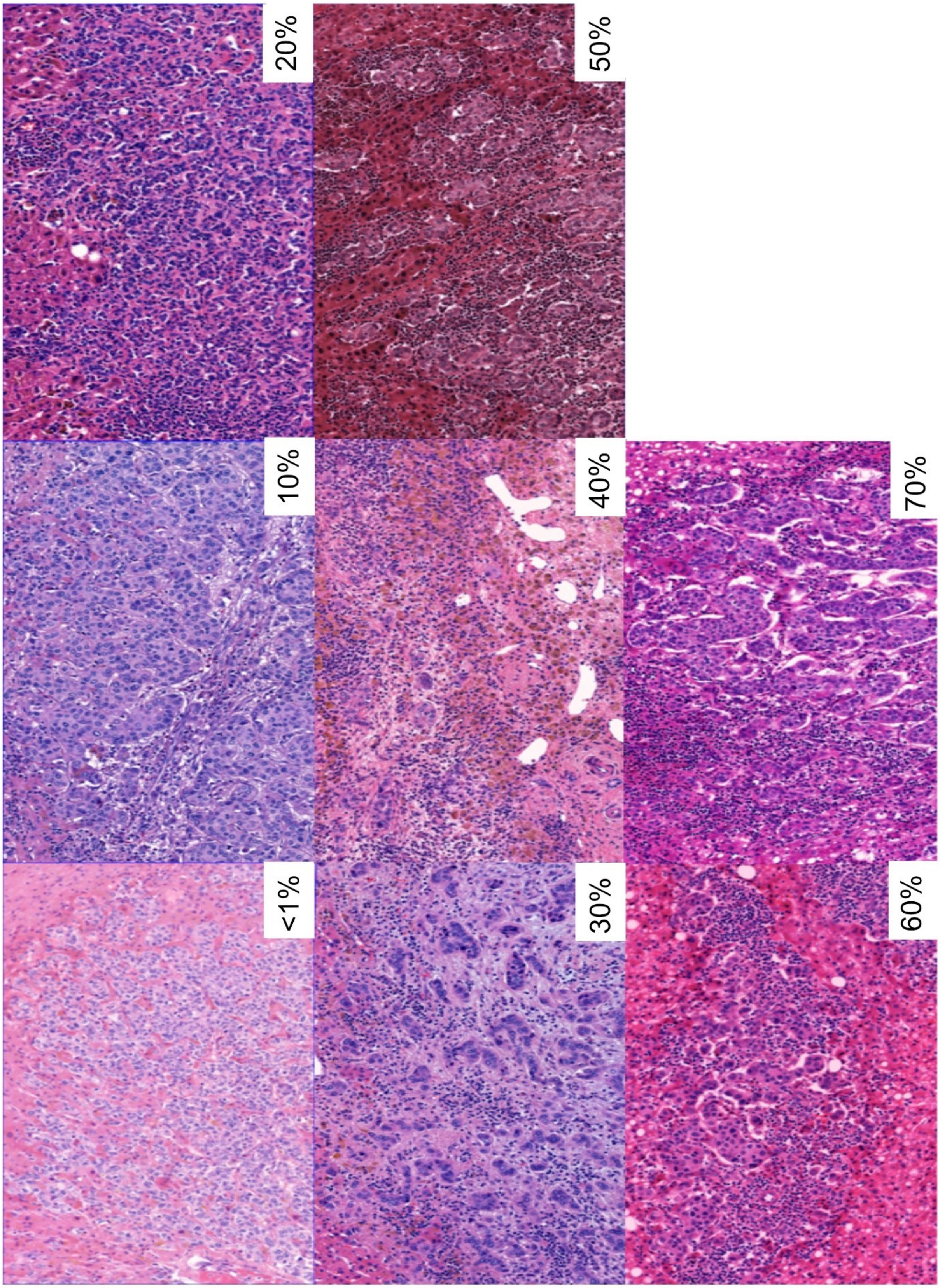
<1%
10%
20%
30%
40%
50%
60%
70%

**Supplementary Figure 2**: Guidelines for TIL assessment. TIL are reported as a percentage of the stroma occupied by lymphocytic cells according to the total area of the stroma in the well-defined area. The final TIL score for each unique BCLM is evaluated as the average of the scores per slide. **a.** Assessment of TIL in d-HGP BCLM. **b.** Assessment of TIL in r-HGP. A r-HGP BCLM with more than 70% of TIL was not observed. Abbreviations: TIL= Tumor Infiltrating Lymphocytes; BCLM= Breast Cancer Liver Metastasis; d-HGP= Desmoplastic; r-HGP= Replacement.

**Supplementary Figures**

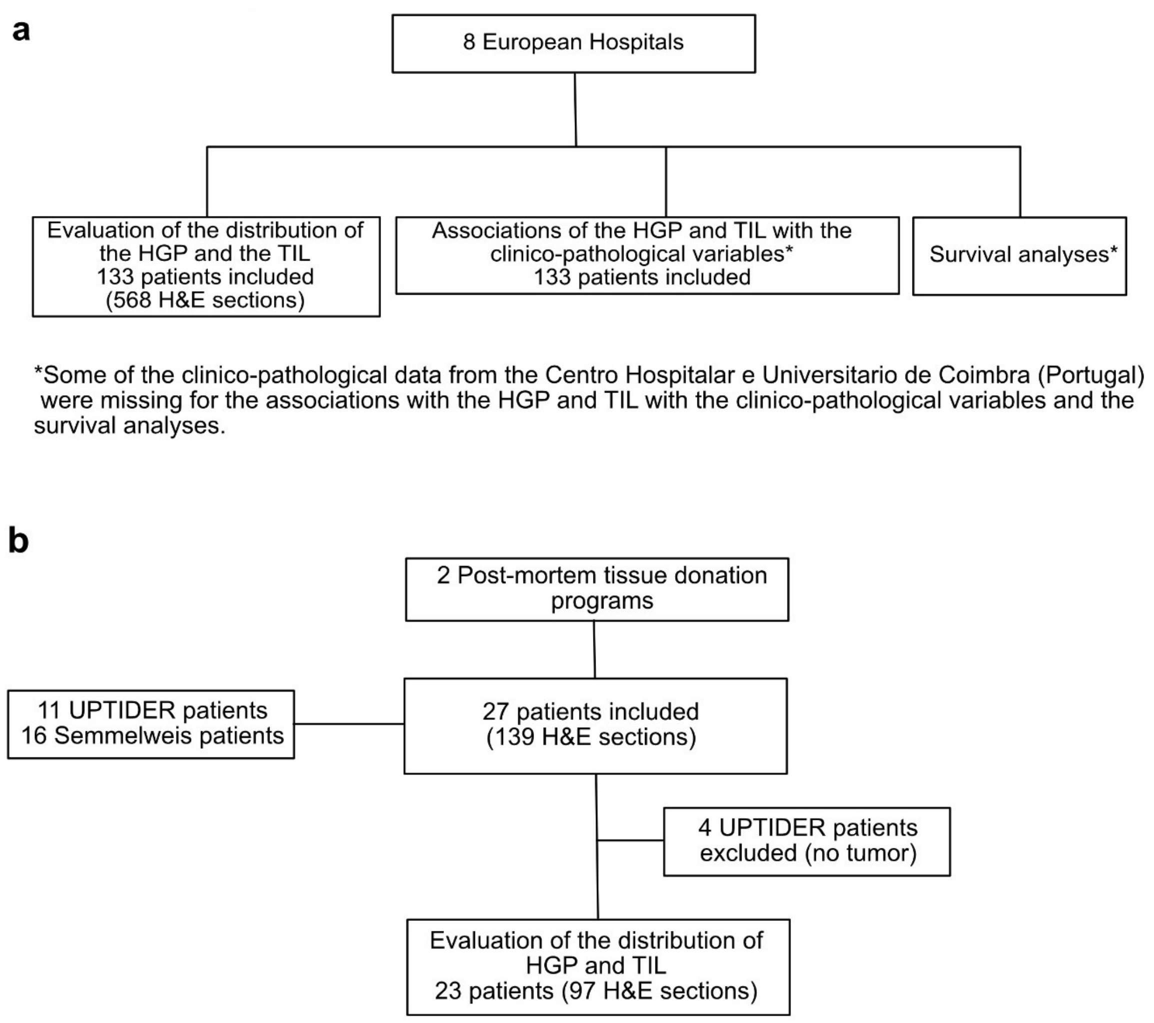


**Supplementary Figure 3: a.** Flowchart of the surgically resected LM cohort (surgical cohort). We included 133 patients (568 H&E sections) from eight European hospitals in the evaluation of the distribution of the HGP and TIL and in the analyses of the association of the HGP and TIL with the clinico-pathological variables. Note that since a majority of the clinico-pathological data from the Centro Hospitalar e Universitario de Coimbra (Portugal) were missing because the patients only got liver surgery in this hospital and were not further treated there, we lost some patients for the regression (associations) and survival analyses. **b.** Flowchart of the post-mortem cohort. We included 27 patients (139 H&E sections) from the UPTIDER (11 patients) and Semmelweis (16 patients) tissue donation programs. However, we had to exclude 4 patients (42 H&E sections) because no tumor or no tumor-liver interface was detected by the histopathological revision. This resulted in 23 patients (97 H&E sections) in this cohort. Abbreviations: TIL= Tumor Infiltrating Lymphocytes; HGP= Histopathological Growth Pattern; H&E= Hematoxylin and Eosin; PFS= Progression Free Survival; OS= Overall Survival.

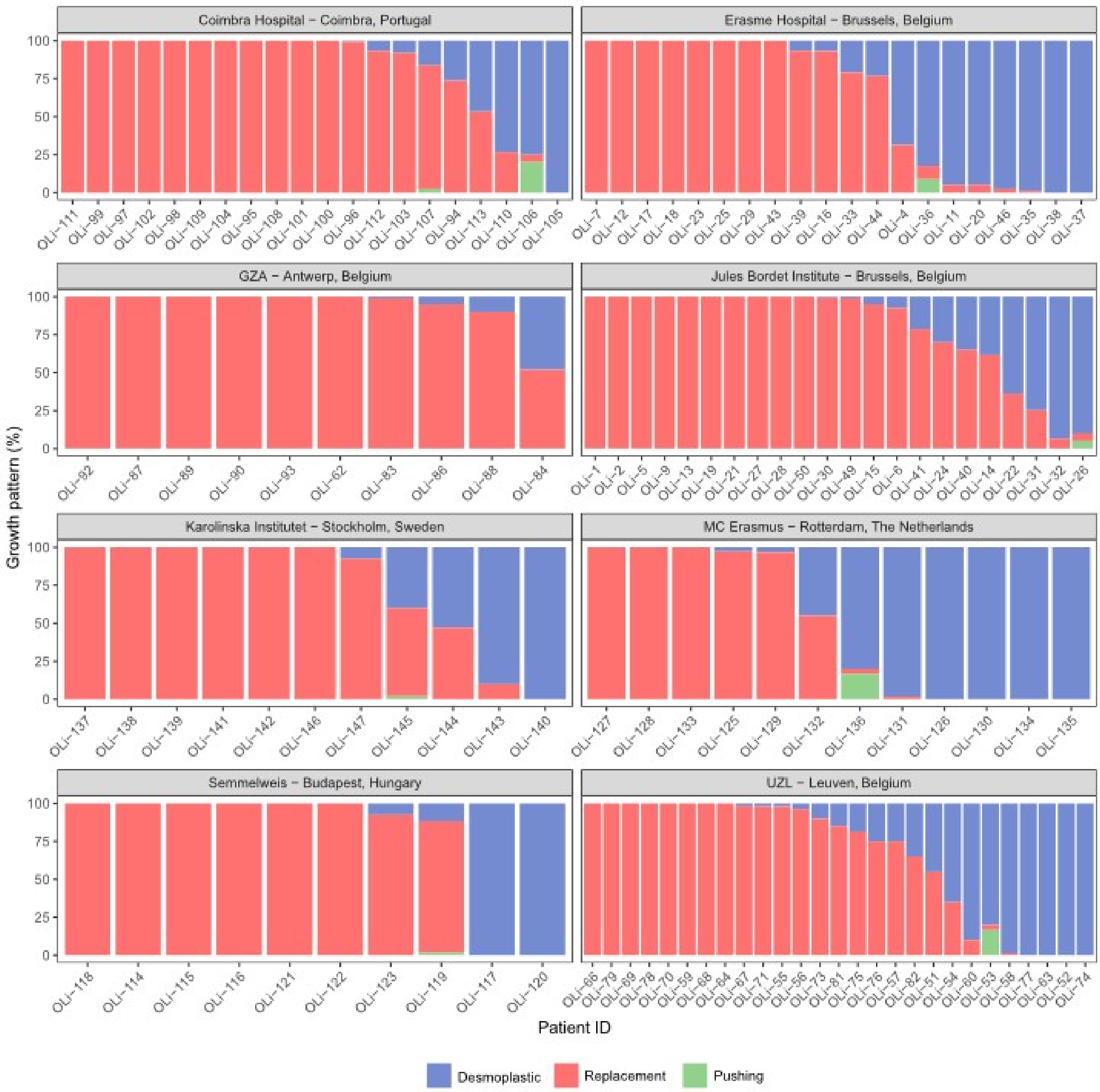


**Supplementary Figure 4: The HGP distribution (%) according to the centers.** y-axis = percentage of growth pattern; x-axis = patient ID. Red= replacement; blue= desmoplastic; green= pushing.

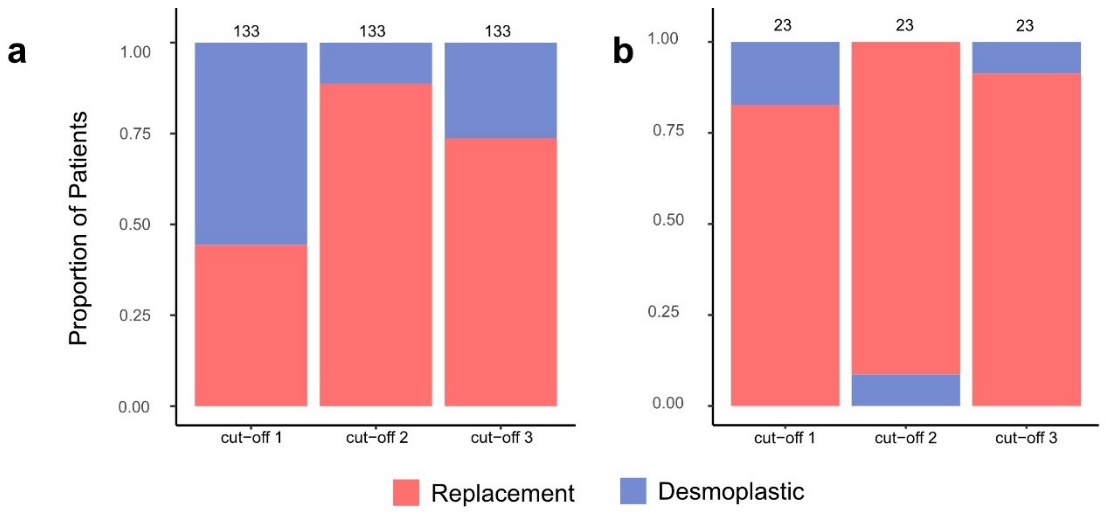


**Supplementary Figure 5: Histopathological Growth Pattern (HGP) distribution in liver metastasis (LM) from breast cancer (BC) patients using different cut-offs (exploratory). a.** Proportion of patients (%, y-axis) per HGP categories according to the three cut-offs (x-axis) in the surgical cohort with 74 (56%) patients with 'any d-HGP' vs 59 (44%) patients with 'pure r-HGP' (cut-off 1); 118 (89%) patients with 'any r-HGP' vs 15 (11%) patients with 'pure d-HGP' (cut-off 2) and 35 (26%) patients with dominant d-HGP vs 98 (74%) patients with dominant r-HGP (cut-off 3). **b.** Proportion of patients (%, y-axis) per HGP categories according to the three cut-offs (x-axis)according to the three cut-offs in the post-mortem cohort with 4 (17%) patients with 'any d-HGP' vs 19 (83%) patients with 'pure r-HGP' (cut-off 1); 21 (91%) patients with 'any r-HGP' vs 2 (9%) with 'pure d-HGP' (cut-off 2) and 21 (91%) with dominant r-HGP vs 2 (9%) with dominant d-HGP (cut-off 3). Red= Replacement; blue= Desmoplastic. Abbreviations: HGP= Histopathological Growth Pattern; BC= Breast Cancer; LM= Liver Metastasis.

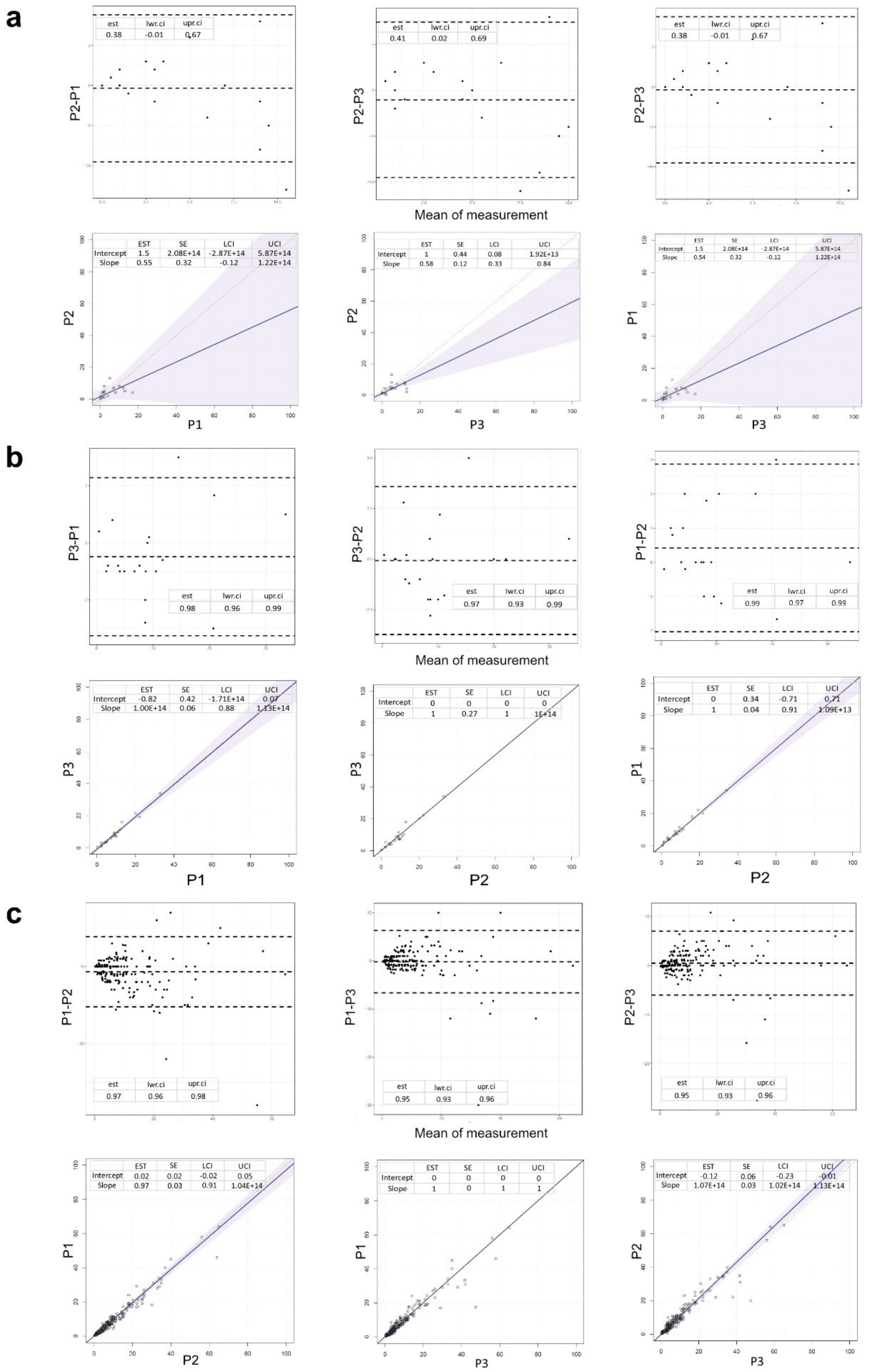


**Supplementary Figure 6: TIL scoring inter-pathologists (P) concordance correlation coefficient (CCC).** **a**. Bland-Altman (above) and Passing-Bablock (below) tests on 20 randomly chosen slides with guidelines used in BC(1) (P2vsP1 CCC=.38; P2vsP3 CCC=.41; P1vsP3 CCC=.38) **b**. Bland-Altman (above)

and Passing-Bablock (below) tests on the same 20 randomly chose slides as on figure 3A with new redefined guidelines (P3vsP1 CCC=.98; P3vsP2 CCC=.97; P1vsP2 CCC=.99) **c.** Bland-Altman (above) and Passing-Bablock (below) tests on 213 slides with new redefined guidelines ((P1vsP2 CCC=.97; P1vsP3 CCC=.95; P2vsP3 CCC=.95). Bland-Altman: y-axis= difference between scores from rater A and rater B; x-axis = mean of measurements. Passing Bablock: y-axis= scores of rater A; x-axis= scores of rater B. Abbreviations: TIL= Tumor Infiltrating Lymphocytes; BC= Breast Cancer; P= Pathologist; CCC= Concordance Correlation Coefficient.

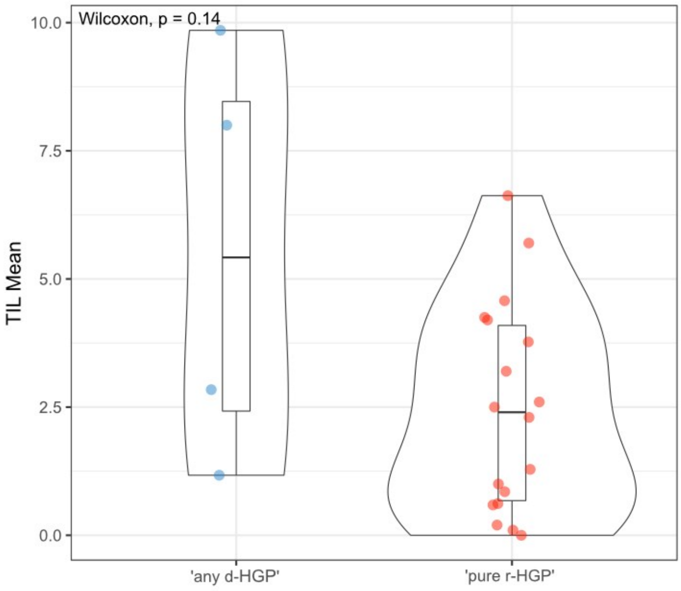


**Supplementary Figure 7: Association between the TIL (%) and the HGP in the post-mortem cohort** (p-value= .14; Wilcoxon-Man Whitney test). Y-axis= Percentage (%) of TIL; x-axis = HGP categories. Blue= Desmoplastic; red= Replacement. Abbreviations: HGP= Histopathological Growth Pattern; TIL= Tumor Infiltrating Lymphocytes; d-HGP= Desmoplastic; r-HGP= Replacement.

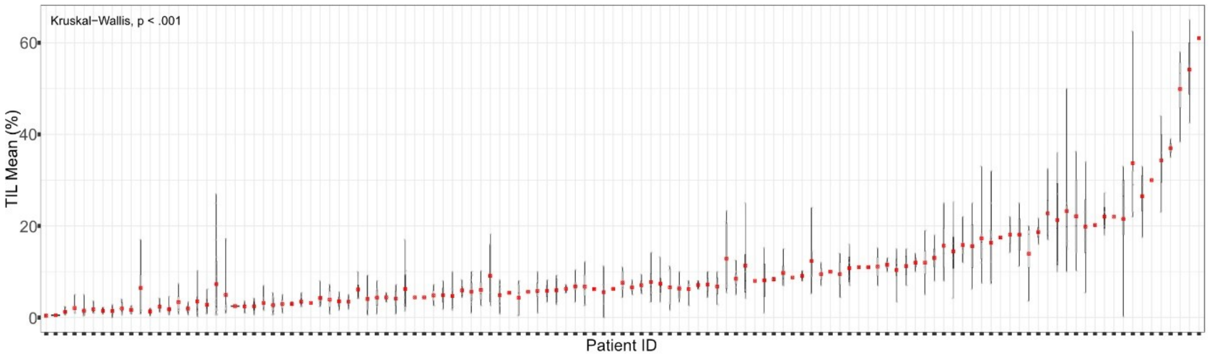


**Supplementary Figure 8: TIL mean inter-slide intra-patient heterogeneity according to the patient ID in the surgical cohort.** TIL heterogeneity in 36 (27%) patients using 10% as difference between two slides and in 76 (57%) patients for a difference of 5% between two slides (p-value<.001; Kruskal-Wallis

test). Y-axis= TIL mean (%); x-axis = Patient ID. Patient ID are ordered by ascending TIL median. The red dots represent the mean. Abbreviations: TIL= Tumor Infiltrating Lymphocytes.

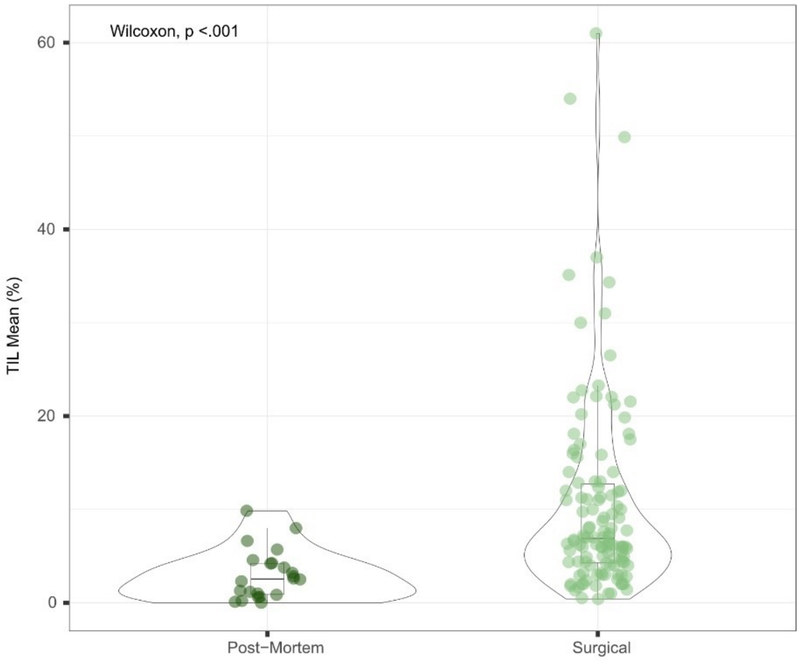


**Supplementary Figure 9**: **Comparison of distribution of TIL in both cohorts.** In the post-mortem cohort (left), median = 2.90% (range: 0.80%-4.61%) and in the surgical cohort (right) a median = 6.68% (range: 4.03%-12.09%). (p-value<.001; Wilcoxon-Mann Whitney Test). Y-axis = TIL mean (%); x-axis = cohorts. Abbreviations: TIL= Tumor Infiltrating Lymphocytes.

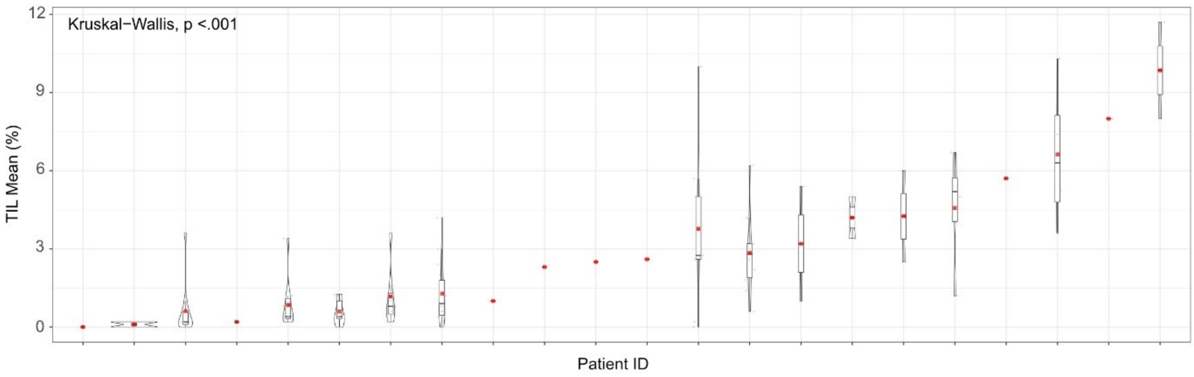


**Supplementary Figure 10**: TIL mean inter-slide intra-patient heterogeneity according to the patient ID in the post-mortem cohort. The TIL median per patient is 1.33% (range: 1%-3.32%). Only 1 (7%) patient showed a TIL inter-slide heterogeneity using 10% as difference between two slides and 4 (28.9%) using 5% (p-value<.001; Kruskal-Wallis test). Y-axis= TIL mean (%); x-axis = Patient ID. Patient ID are ordered by ascending TIL median. The red dots represent the mean. Abbreviations: TIL= Tumor Infiltrating Lymphocytes.

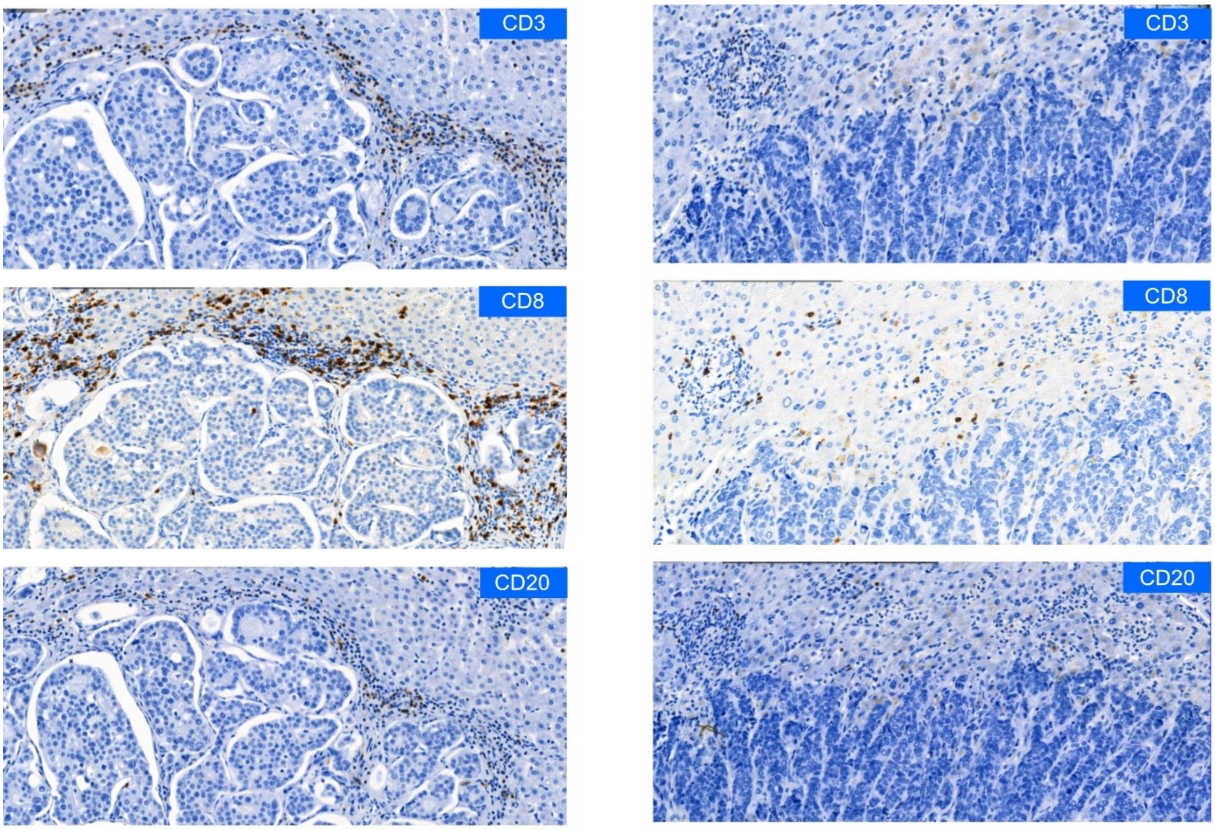


**Supplementary Figure 11**: **Immunohistochemistry (IHC) using CD3 (top), CD8 (middle), CD20 (bottom) as markers on a BCLM**. Desmoplastic HGP (left) and on a LM with replacement HGP (right) illustrating a lower immune infiltrate in LM with r-HGP as compared to LM with d-HGP. Abbreviations: HGP= Histopathological Growth Pattern; BCLM= Breast Cancer Liver Metastasis; LM= Liver Metastasis.

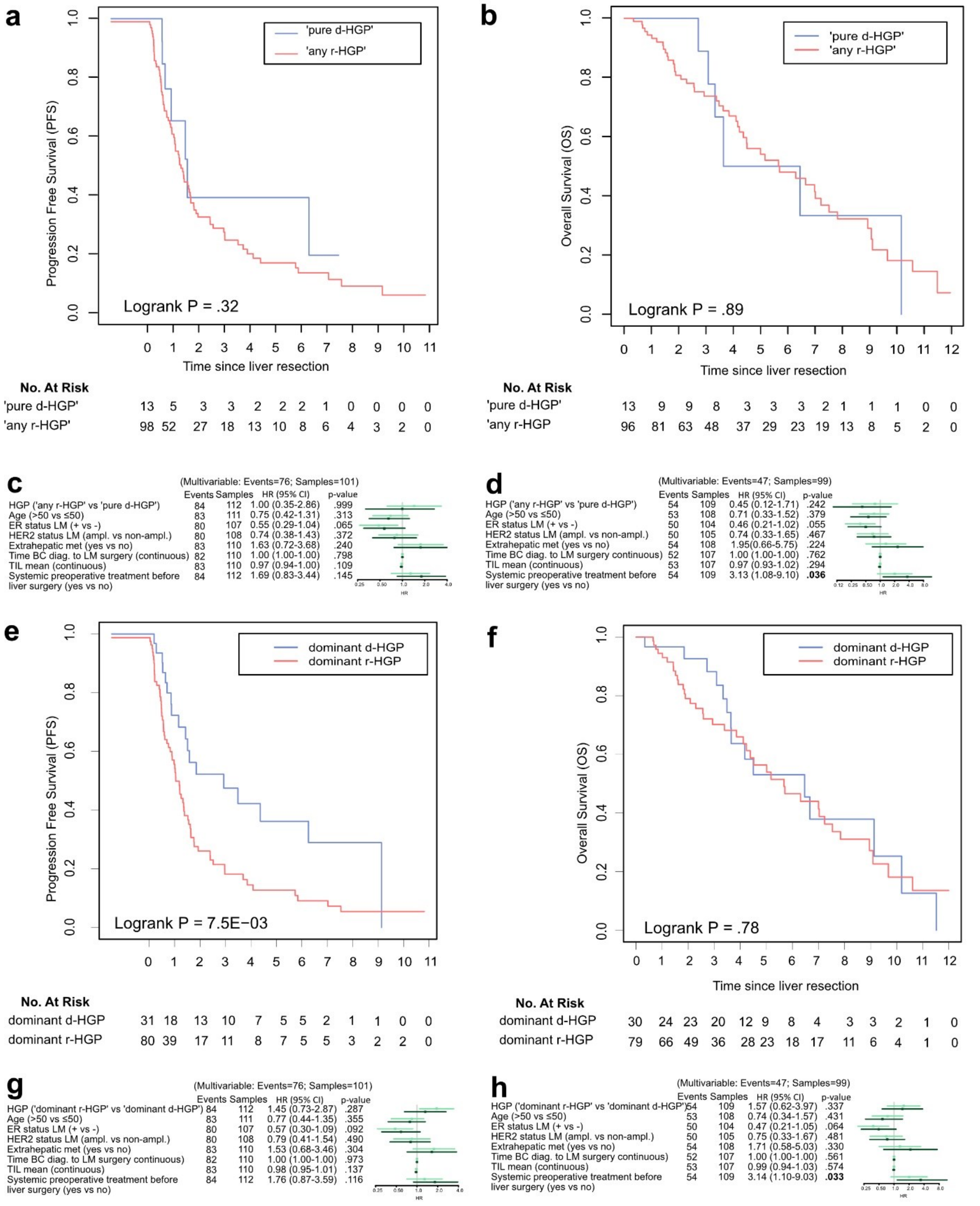


**Supplementary Figure 12: Survival analyses using two other cut-offs (exploratory). A-D. Survival analyses using the cut-off 'pure d-HGP' versus 'any r-HGP'. a-b.** Survival curves (Kaplan-Meier) using the 'any r-HGP' versus 'pure d-HGP' categories. The horizontal axis (x-axis) represents time in years, and the vertical axis (y-axis) shows the probability of surviving. **a.** Association between Progression Free Survival (PFS) and the HGP categories (Logrank p = .32). Start event is the first liver resection; end event is the progression or death. **b.** Association between Overall Survival (OS) and the HGP categories (Logrank p = .89). Start event is the first liver resection; end event is the death or last follow-up. **c.**

Univariable and multivariable Cox regression analyses for PFS. **d.** Univariable and multivariable Cox regression analyses for OS. Only the systemic preoperative treatment before liver surgery is significantly associated with a worse PFS (p-value=.0.36). **e-h. Survival analyses using the cut-off 'dominant d-HGP' versus 'dominant r-HGP'. e-f.** Survival curves (Kaplan-Meier) using the dominant r-HGP versus dominant d-HGP categories. The horizontal axis (x-axis) represents time in years, and the vertical axis (y-axis) shows the probability of surviving. **e.** Association between Progression Free Survival (PFS) and the HGP categories (Logrank p = 7.5e-3). Start event is the first liver resection; end event is the progression or death. **f.** Association between Overall Survival (OS) and the HGP categories (Logrank p = .78). Start event is the first liver resection; end event is the death or last follow-up. **g.** Univariable and multivariable Cox regression analyses for PFS. **h.** Univariable and multivariable Cox regression analyses for OS. The multivariable model included: HGP ('pure r-HGP' vs 'any d-HGP), age at diagnosis (>50 vs ≤50), presence of extrahepatic metastasis (yes vs no), time between BC diagnosis (continuous, per one-year increase) and LM surgery, TIL (continuous, per one percent increase) and systemic preoperative treatment before liver surgery (yes vs no). Blue= Desmoplastic; red= Replacement; light green= univariable; dark green= multivariable. Abbreviations: HR= Hazard Ratio; CI= Confidence Interval; HGP= Histopathological Growth Pattern; d-HGP= Desmoplastic; r-HGP= Replacement); met= Metastasis; BC= Breast Cancer; LM= Liver Metastasis; TIL= Tumor Infiltrating Lymphocytes; diag.= Diagnosis; ampl. = Amplified; non-ampl.= Non-amplified; PFS= Progression Free Survival; OS= Overall Survival.

**Supplementary tables**

**Supplementary Table 1**: Clinico-pathological characteristics of the surgical cohort using the following categories: ‘any r-HGP’ versus ‘pure d-HGP’*

| **Clinicopathological characteristics of the primary disease** | | | | | | |
|---|---|---|---|---|---|---|
| | **n** | **‘pure d-HGP'** | **‘any r-HGP'** | **OR** | **CI 95%** | **p** |
| **Menopausal status (post- vs pre-menopausal)** | | | | 0.65 | 0.18-2.35 | 0.509 |
| post-menopausal | 42 | 6 (46.2) | 36 (40.0) | | | |
| pre-menopausal | 61 | 7 (53.8) | 54 (60.0) | | | |
| Missing | 30 | 2 | 28 | | | |
| **Age (>50 vs ≤50 years)** | | | | 0.36 | 0.10-1.18 | 0.092 |
| ≤ 50 | 68 | 8 (57.1) | 60 (65.2) | | | |
| >50 | 38 | 6 (42.9) | 32 (34.8) | | | |
| Missing | 27 | 1 | 26 | | | |
| **cT (>1 vs 1)** | | | | 0.35 | 0.03-1.84 | 0.239 |
| 1 | 20 | 1 (7.1) | 19 (22.1) | | | |
| 2 | 52 | 6 (42.9) | 46 (53.5) | | | |
| 3 | 17 | 5 (35.7) | 12 (13.9) | | | |
| 4 | 11 | 2 (14.3) | 9 (10.5) | | | |
| Missing | 33 | 1 | 32 | | | |
| **cN (≥1 vs 0)** | | | | 0.88 | 0.26-3.01 | 0.840 |
| 0 | 46 | 7 (50.0) | 39 (50.0) | | | |
| 1 | 37 | 5 (35.7) | 32 (41.0) | | | |
| 2 | 8 | 2 (14.3) | 6 (7.7) | | | |
| 3c | 1 | 0 (0.0) | 1 (1.3) | | | |
| Missing | 41 | 1 | 40 | | | |
| **cM (1 vs 0)** | | | | 0.57 | 1.15-2.18 | 0.401 |
| 0 | 57 | 8 (61.5) | 49 (71.0) | | | |
| 1 | 25 | 5 (38.5) | 20 (29.0) | | | |
| Missing | 51 | 2 | 49 | | | |
| **pN (1 vs 0)** | | | | 2.44 | 0.46-14.56 | 0.289 |
| 0 | 26 | 4 (66.7) | 22 (36.1) | | | |
| 1 | 41 | 2 (33.3) | 39 (63.9) | | | |
| Missing | 46 | 9 | 57 | | | |
| **Histological subtype (ILC vs NST)** | | | | 1.34 | 0.13-7.39 | 0.766 |
| invasive ductal adenocarcinoma (NST) | 106 | 13 (92.9) | 93 (84.5) | | | |
| invasive lobular adenocarcinoma (ILC) | 17 | 1 (7.1) | 16 (14.5) | | | |
| Missing | 10 | 1 | 9 | | | |
| **Histological grade (2 and 3 vs 1)** | | | | 0.19 | 0.01-1.78 | 0.173 |
| 1 | 15 | 0 (0.00) | 15 (20.00) | | | |
| 2 | 46 | 8 (72.8) | 38 (50.7) | | | |
| 3 | 25 | 3 (27.2) | 22 (29.3) | | | |
| Missing | 47 | 4 | 43 | | | |
| **Laterality (right vs left)** | | | | 0.82 | 0.24-2.78 | 0.738 |

| | | | | | | |
|---|---|---|---|---|---|---|
| bilateral | 8 | 1 (16.7) | 7 (12.1) | | | |
| left | 54 | 5 (83.3) | 49 (84.5) | | | |
| right | 2 | 0 (0.0) | 2 (3.4) | | | |
| Missing | 69 | 9 | 60 | | | |
| **ER-status (positive vs negative)** | | | | 1.43 | 0.25-6.23 | 0.658 |
| negative | 18 | 2 (13.3) | 16 (16.0) | | | |
| positive | 97 | 13 (86.7) | 84 (84.0) | | | |
| Missing | 18 | 0 | 18 | | | |
| **HER2-status (amplified vs non-amplified)** | | | | 0.35 | 0.10-1.33 | 0.119 |
| non-amplified | 89 | 9 (64.3) | 80 (86.0) | | | |
| amplified | 18 | 5 (35.7) | 13 (14.0) | | | |
| Missing | 26 | 1 | 25 | | | |
| **Neoadjuvant chemotherapy (yes vs no)** | | | | 0.47 | 0.12-1.84 | 0.272 |
| no | 76 | 8 (61.5) | 68 (75.6) | | | |
| yes | 27 | 5 (38.5) | 22 (24.4) | | | |
| Missing | 30 | 2 | 28 | | | |
| **Clinicopathological characteristics of the liver metastasis** | | | | | | |
| **ER-status (positive vs negative)** | | | | 0.75 | 0.14-2.90 | 0.696 |
| negative | 28 | 1 (8.3) | 27 (23.7) | | | |
| positive | 98 | 11 (91.7) | 87 (76.3) | | | |
| Missing | 7 | 3 | 4 | | | |
| **HER2-status (amplified vs non-amplified)** | | | | 0.87 | 0.23-4.08 | 0.845 |
| non-amplified | 89 | 9 (75.0) | 91 (79.1) | | | |
| amplified | 27 | 3 (25.0) | 24 (20.9) | | | |
| Missing | 6 | 3 | 3 | | | |
| **Extrahepatic metastasis (yes vs no)** | | | | 3.00 | 0.52-32.43 | 0.237 |
| no | 94 | 13 (92.9) | 81 (83.5) | | | |
| yes | 17 | 1 (7.1) | 16 (16.5) | | | |
| Missing | 22 | 1 | 21 | | | |
| **Time between BC diagnosis and liver surgery (continuous)** | | | | 1.00 | 1.00-1.00 | 0.103 |
| <1month | 27 | 7 (50.0) | 20 (20.6) | | | |
| <1year | 8 | 0 (0.0) | 8 (8.2) | | | |
| <2 years | 11 | 3 (21.4) | 8 (8.2) | | | |
| ≥2years | 65 | 4 (28.6) | 61 (62.9) | | | |
| Missing | 22 | 1 | 21 | | | |
| **Systemic preoperative treatment before liver surgery (yes vs no)** | | | | 0.49 | 0.11-1.76 | 0.286 |
| no | 34 | 3 (20.0) | 31 (26.3) | | | |
| yes | 99 | 12 (80.0) | 87 (73.7) | | | |
| **First site of progression (liver vs other)** | | | | 0.77 | 0.13-8.13 | 0.794 |
| liver (only) | 86 | 8 (88.9) | 78 (84.8) | | | |
| liver and bone | 2 | 0 (0.0) | 2 (2.1) | | | |
| liver, bone and brain | 1 | 0 (0.0) | 1 (1.1) | | | |
| other | 12 | 1 (11.1) | 11 (12.0) | | | |

| | | | | | | |
|---|---|---|---|---|---|---|
| unknown | 32 | 6 | 26 | | | |

Regarding logistic regressions, the outcome is the HGP with 'any r-HGP' as an event. For each variable, the reference is given on the right side of its label, and an OR>1 indicates a positive association with 'any r-HGP'. Abbreviations: BC= Breast Cancer; T = primary tumor; N = regional lymph node; M = distant metastasis; c = clinical; CI=Confidence Interval; ER= Estrogen Receptor; HER2 = Human Epidermal Growth Factor Receptor-2; NST= No Special Type; ILC= Invasive Lobular Carcinoma; OR= Odds Ratio.

**Supplementary Table 2**: Clinico-pathological characteristics of surgical cohort using the following categories: 'dominant r-HGP' versus 'dominant d-HGP'

| **Clinicopathological characteristics of the primary disease** | | | | | | |
|---|---|---|---|---|---|---|
| | **n** | **'dominant d-HGP'** | **'dominant r-HGP'** | **OR** | **CI 95%** | **p** |
| **Menopausal status (post- vs pre-menopausal)** | | | | 1.26 | 0.51-3.25 | 0.616 |
| post-menopausal | 42 | 10 (34.5) | 32 (43.2) | | | |
| pre-menopausal | 61 | 19 (65.5) | 42 (56.8) | | | |
| Missing | 30 | 6 | 24 | | | |
| **Age (>50 vs ≤50 years)** | | | | 0.68 | 0.29-1.61 | 0.372 |
| ≤ 50 | 68 | 18 (60.0) | 50 (65.8) | | | |
| >50 | 38 | 12 (40.0) | 26 (34.2) | | | |
| Missing | 27 | 5 | 22 | | | |
| **cT (>1 vs 1)** | | | | 1.73 | 0.56-5.24 | 0.336 |
| 1 | 20 | 7 (26.9) | 13 (17.6) | | | |
| 2 | 52 | 11 (42.3) | 41 (55.4) | | | |
| 3 | 17 | 6 (23.1) | 11 (14.9) | | | |
| 4 | 11 | 2 (3.8) | 9 (9.5) | | | |
| Missing | 33 | 9 | 24 | | | |
| **cN (≥1 vs 0)** | | | | 1.54 | 0.58-4.19 | 0.385 |
| 0 | 46 | 15 (57.7) | 31 (47.0) | | | |
| 1 | 37 | 9 (34.6) | 28 (42.4) | | | |
| 2 | 8 | 2 (7.7) | 6 (9.1) | | | |
| 3c | 1 | 0 (0.0) | 1 (1.5) | | | |
| Missing | 41 | 9 | 32 | | | |
| **cM (1 vs 0)** | | | | 0.46 | 0.16-1.31 | 0.144 |
| 0 | 57 | 14 (56.0) | 43 (75.4) | | | |
| 1 | 25 | 11 (44.0) | 14 (24.6) | | | |
| Missing | 51 | 10 | 41 | | | |
| **pN (1 vs 0)** | | | | 4.22 | 1.32-14.60 | **0.015** |
| 0 | 26 | 11 (68.8) | 15 (29.4) | | | |
| 1 | 41 | 5 (31.2) | 36 (70.6) | | | |
| Missing | 66 | 19 | 47 | | | |
| **Histological subtype (NST vs ILC)** | | | | 0.83 | 0.20-2.85 | 0.780 |
| invasive ductal adenocarcinoma (NST) | 106 | 30 (90.9) | 76 (83.5) | | | |
| invasive lobular adenocarcinoma (ILC) | 17 | 3 (9.1) | 14 (15.4) | | | |
| Missing | 10 | 2 | 8 | | | |
| **Histological grade (2 and 3 vs 1)** | | | | 0.80 | 0.21-2.66 | 0.721 |

| | | | | | | |
|---|---|---|---|---|---|---|
| 1 | 15 | 4 (15.4) | 11 (18.3) | | | |
| 2 | 46 | 16 (61.5) | 30 (50.0) | | | |
| 3 | 25 | 6 (23.1) | 19 (31.7) | | | |
| Missing | 47 | 9 | 38 | | | |
| **Laterality (right vs left)** | | | | 1.60 | 0.30-7.81 | 0.565 |
| bilateral | 8 | 3 (15.8) | 5 (11.1) | | | |
| left | 54 | 16 (84.2) | 38 (84.4) | | | |
| right | 2 | 0 (0.0) | 2 (4.4) | | | |
| Missing | 69 | 16 | 53 | | | |
| **ER-status (positive vs negative)** | | | | 1.26 | 0.40-3.71 | 0.677 |
| negative | 18 | 6 (17.6) | 12 (14.8) | | | |
| positive | 97 | 28 (82.4) | 69 (85.2) | | | |
| Missing | 18 | 1 | 17 | | | |
| **HER2-status (amplified vs non-amplified)** | | | | 0.36 | 0.12-1.05 | 0.061 |
| non-amplified | 89 | 24 (72.7) | 65 (87.8) | | | |
| amplified | 18 | 9 (27.3) | 9 (12.2) | | | |
| Missing | 26 | 2 | 24 | | | |
| **Neoadjuvant chemotherapy (yes vs no)** | | | | 0.40 | 0.14-1.14 | 0.087 |
| no | 76 | 18 (60.0) | 58 (79.5) | | | |
| yes | 27 | 12 (40.0) | 15 (20.5) | | | |
| Missing | 30 | 5 | 25 | | | |
| **Clinicopathological characteristics of the liver metastasis** | | | | | | |
| **ER-status (positive vs negative)** | | | | 0.81 | 0.29-2.08 | 0.667 |
| negative | 28 | 6 (19.4) | 22 (23.2) | | | |
| positive | 98 | 25 (80.6) | 73 (76.8) | | | |
| Missing | 7 | 4 | 3 | | | |
| **HER2-status (amplified vs non-amplified)** | | | | 0.46 | 0.18-1.17 | 0.102 |
| non-amplified | 100 | 20 (64.5) | 80 (83.3) | | | |
| amplified | 27 | 11 (35.5) | 16 (16.7) | | | |
| Missing | 6 | 4 | 2 | | | |
| **Extrahepatic metastasis (yes vs no)** | | | | 2.44 | 0.65-11.63 | 0.192 |
| no | 94 | 29 (90.6) | 65 (82.3) | | | |
| yes | 17 | 3 (9.4) | 14 (17.7) | | | |
| Missing | 22 | 3 | 19 | | | |
| **Time between BC diagnosis and liver surgery (continuous)** | | | | 1.00 | 1.00-1.00 | **0.023** |
| <1month | 27 | 12 (38.7) | 15 (18.8) | | | |
| <1year | 8 | 1 (3.2) | 7 (8.8) | | | |
| <2 years | 11 | 4 (12.9) | 7 (8.8) | | | |
| ≥2years | 65 | 14 (45.2) | 51 (63.8) | | | |
| Missing | 22 | 4 | 18 | | | |
| **Systemic preoperative treatment before liver surgery (yes vs no)** | | | | 0.29 | 0.08-0.87 | **0.026** |
| no | 34 | 4 (11.4) | 30 (30.6) | | | |
| yes | 99 | 31 (88.6) | 68 (69.4) | | | |
| **First site of progression (liver vs other)** | | | | 1.70 | 0.43-9.56 | 0.471 |

| | | | |
|---|---|---|---|
| liver (only) | 86 | 23 (88.5) | 63 (84.0) |
| liver and bone | 2 | 0 (0.0) | 2 (2.7) |
| liver, bone and brain | 1 | 0 (0.0) | 1 (1.3) |
| other | 12 | 3 (11.5) | 9 (12.0) |
| unknown | 32 | 9 | 23 |

Regarding logistic regressions, the outcome is the HGP with 'dominant r-HGP' as an event. For each variable, the reference is given on the right side of its label, and an OR>1 indicates a positive association with 'dominant r-HGP'. Abbreviations: BC= Breast Cancer; T= primary tumor; N= regional lymph node; M= distant metastasis; c= clinical; p= pathological; CI=Confidence Interval; ER= Estrogen Receptor; HER2 = Human Epidermal Growth Factor Receptor-2; NST= No Special Type; ILC= Invasive Lobular Carcinoma; OR= Odds Ratio.

**Supplementary Table 3:** Clinico-pathological characteristics of the post-mortem cohort using the following categories: 'pure r-HGP' versus 'any d-HGP'

| **Clinicopathological characteristics of the primary disease** | | | |
|---|---|---|---|
| | **n** | **'pure r-HGP'** | **'any d-HGP'** |
| **age** | | | |
| ≤ 50 | 8 | 7 (36.8) | 1 (25.0) |
| >50 | 15 | 12 (63.2) | 3 (75.0) |
| Missing | 0 | 0 | 0 |
| **cT** | | | |
| 1 | 3 | 3 (24.6) | 1 (33.3) |
| 2 | 7 | 6 (50.0) | 1 (33.3) |
| 3 | 1 | 1 (8.3) | 0 (00.0) |
| 4 | 3 | 2 (16.6) | 1 (33.3) |
| Missing | 8 | 7 | 1 |
| **cN** | | | |
| 0 | 6 | 4 (33.3) | 2 (50.0) |
| 1 | 6 | 4 (33.3) | 2 (50.0) |
| 1a | 1 | 1 (8.3) | 0 (00.0) |
| 1b | 1 | 1 (8.3) | 0 (00.0) |
| 2 | 1 | 1 (8.3) | 0 (00.0) |
| 3 | 1 | 1 (8.3) | 0 (00.0) |
| Missing | 7 | 7 | 0 |
| **cM** | | | |
| 0 | 10 | 7 (53.8) | 3 (75.0) |
| 1 | 7 | 6 (46.2) | 1 (25.0) |
| Missing | 6 | 6 | 0 |
| **Histological subtype (primary)** | | | |
| Invasive BC of no special type (NST) | 19 | 16 (84.2) | 3 (75.0) |
| Invasive lobular carcinoma (ILC) | 4 | 3 (15.8) | 1 (25.0) |
| Missing | 0 | 0 | 0 |
| **Laterality (primary)** | | | |
| left | 8 | 6 (75.0) | 2 (50.0) |
| right | 2 | 2 (25.0) | 0 (50.0) |
| Missing | 13 | 11 | 2 |

| | | | |
|---|---|---|---|
| **ER (primary)** | | | |
| negative | 7 | 7(36.8) | 0 (0.00) |
| positive | 16 | 12 (63.2) | 4 (100.0) |
| Missing | 0 | 0 | 0 |
| **HER2 (primary)** | | | |
| negative | 16 | 14 (51.8) | 2 (66.7) |
| positive | 4 | 3 (48.2) | 1(33.3) |
| Missing | 3 | 2 | 1 |

Abbreviations: BC= Breast Cancer; T= primary tumor; N= regional lymph node; M= distant metastasis; c= Clinical; p= Pathological; ER= Estrogen Receptor; HER2 = Human Epidermal Growth Factor Receptor-2; NST= No Special Type; ILC= Invasive Lobular Carcinoma.

**Supplementary Table 4:** Systemic preoperative treatment before liver surgery using the following categories: 'pure r-HGP' versus 'any d-HGP'

| | | **n** | **'any d-HGP'** | **'pure r-HGP'** |
|---|---|---|---|---|
| **Systemic preoperative treatment before liver surgery** | | | | |
| | no | 34 | 13 (21.6) | 21 (39.0) |
| | yes | 99 | 61 (78.4) | 38 (61.0) |
| **CDK4/6 inhibitors** | | | | |
| | no | 84 | 45 (82.5) | 39 (88.6) |
| | yes | 15 | 10 (17.6) | 5 (11.4) |
| | missing | 32 | 17 | 15 |
| **SERM** | | | | |
| | no | 93 | 54 (94.7) | 39 (88.6) |
| | yes | 8 | 3 (5.3) | 5 (11.4) |
| | missing | 32 | 17 | 15 |
| **Aromatase Inhibitors** | | | | |
| | no | 69 | 35 (62.5) | 34 (79.1) |
| | yes | 30 | 21 (37.5) | 9 (20.9) |
| | missing | 34 | 18 | 16 |
| **SERD** | | | | |
| | no | 94 | 54 (94.7) | 40 (90.9) |
| | yes | 7 | 3 (5.3) | 4 (9.1) |
| | missing | 32 | 17 | 15 |
| **mTOR inhibitor** | | | | |
| | no | 96 | 54 (94.7) | 42 (77.3) |
| | yes | 5 | 3 (5.3) | 2 (22.7) |
| | missing | 32 | 17 | 15 |
| **Other chemo** | | | | |
| | no | 63 | 40 (70.2) | 23 (52.3) |
| | yes | 38 | 17 (29.8) | 21 (47.7) |
| | missing | 32 | 17 | 15 |
| **Taxanes** | | | | |
| | no | 63 | 34 (59.7) | 29 (65.9) |
| | yes | 38 | 23 (40.3) | 15 (34.1) |

| | | | | |
|---|---|---|---|---|
| | missing | 32 | 17 | 15 |
| **Anti-HER2 Ab** | | | | |
| | no | 82 | 45 (78.9) | 37 (84.1) |
| | yes | 19 | 12 (21.1) | 7 (15.9) |
| | missing | 32 | 17 | 15 |
| **ADC anti-HER2** | | | | |
| | no | 101 | 57 (100.0) | 44 (100.0) |
| | missing | 32 | 17 | 15 |
| **Tyrosine kinase inhibitor** | | | | |
| | no | 98 | 55 (96.5) | 43 (97.7) |
| | yes | 3 | 2 (3.5) | 1 (2.3) |
| | missing | 32 | 17 | 15 |
| **Anti-VEGF** | | | | |
| | no | 99 | 55 (96.5) | 44 (100.0) |
| | yes | 2 | 2 (3.3) | 0 (0.0) |
| | missing | 32 | 17 | 15 |
| **Ovarian function suppressor** | | | | |
| | no | 96 | 52 (91.2) | 44 100.0) |
| | yes | 5 | 5 (8.8) | 0 (0.0) |
| | missing | 32 | 17 | 15 |
| **Denosumab** | | | | |
| | no | 95 | 55 (96.5) | 40 (90.9) |
| | yes | 6 | 2 (3.5) | 4 (9.1) |
| | missing | 32 | 17 | 15 |
| **Other treatment** | | | | |
| | no | 96 | 53 (93.0) | 43 (97.7) |
| | yes | 5 | 4 (7.0) | 1 (2.3) |
| | missing | 32 | 17 | 15 |

Abbreviations: CDK4/6 = Cycline dependent kinase 4/6; SERM = Selective Estrogen Receptor Modulator; SERD = Selective Estrogen Receptor Degrader/Downregulator; mTOR = Mammalian Target of Rapamycin; HER2 = Human Epidermal Growth Factor Receptor 2; ab = Antibody; ADC = Antibody-Drug Conjugate; VEGF = Vascular Endothelial Growth Factor.

**Supplementary Table 5:** Clinico-pathological characteristics of the treatment naïve patients before liver surgery using the following categories: 'pure r-HGP' versus 'any d-HGP'

| **Clinicopathological characteristics of the primary disease** | | | |
|---|---|---|---|
| | **n** | **'any d-HGP'** | **'pure r-HGP'** |
| **Menopausal status (post- vs pre-menopausal)** | | | |
| **post-menopausal** | 10 | 4 (44.4) | 6 (42.8) |
| **pre-menopausal** | 13 | 5 (55.6) | 8 (57.2) |
| **Missing** | 11 | 4 | 7 |
| **Age (>50 vs ≤50 years)** | | | |
| **≤ 50** | 16 | 5 (50.0) | 11 (73.4) |
| **>50** | 9 | 5 (50.0) | 4 (26.6) |
| **Missing** | 9 | 3 | 6 |
| **cT (>1 vs 1)** | | | |
| **1** | 6 | 3 (27.3) | 3 (20.0) |

| | | | |
|---|---|---|---|
| **2** | 12 | 5 (45.4) | 7 (46.7) |
| **3** | 7 | 3 (27.3) | 4 (26.7) |
| **4** | 1 | 0 (0.0) | 1 (6.6) |
| **Missing** | 8 | 2 | 6 |
| **cN (≥1 vs 0)** | | | |
| **0** | 12 | 6 (54.6) | 6 (40.0) |
| **1** | 10 | 4 (36.4) | 6 (40.0) |
| **2** | 4 | 1 (9.00) | 3 (20.0) |
| **3c** | 7 | 1 | 6 |
| **Missing** | | | |
| **cM (1 vs 0)** | | | |
| **0** | 17 | 9 (100.0) | 8 (88.9) |
| **1** | 1 | 0 (0.0) | 1 (12.1) |
| **Missing** | 16 | 4 | 12 |
| **pN (1 vs 0)** | | | |
| **0** | 9 | 5 (62.5) | 4 (44.4) |
| **1** | 8 | 3 (37.5) | 5 (55.6) |
| **Missing** | 17 | 4 | 13 |
| **Histological subtype (ILC vs NST)** | | | |
| **invasive ductal adenocarcinoma (NST)** | 29 | 12 (100.0) | 17 (85.0) |
| **invasive lobular adenocarcinoma (ILC)** | 3 | 0 (0.0) | 3 (15.0) |
| **Missing** | 2 | 1 | 1 |
| **Histological grade (2 and 3 vs 1)** | | | |
| **1** | 5 | 1 (11.1) | 4 (33.3) |
| **2** | 12 | 5 (55.6) | 7 (58.3) |
| **3** | 4 | 3 (33.3) | 1 (8.4) |
| **Missing** | 13 | 4 | 9 |
| **Laterality (right vs left)** | | | |
| **left** | 14 | 7 (70.0) | 7 (46.7) |
| **right** | 11 | 3 (30.0) | 8 (53.3) |
| **Missing** | 9 | 3 | 6 |
| **ER-status (positive vs negative)** | | | |
| **negative** | 3 | 2 (18.2) | 1 (6.3) |
| **positive** | 24 | 9 (81.8) | 15 (93.7) |
| **Missing** | 7 | 2 | 5 |
| **HER2-status (amplified vs non-amplified)** | | | |
| **non-amplified** | 22 | 9 (100.0) | 13 (86.7) |
| **amplified** | 2 | 0 (0.00) | 2 (13.3) |
| **Missing** | 10 | 4 | 6 |
| **Neoadjuvant chemotherapy (yes vs no)** | | | |
| **no** | 18 | 8 (88.9) | 10 (90.1) |
| **yes** | 2 | 1 (11.1) | 1 (9.9) |
| **Missing** | 14 | 4 | 10 |
| **Clinicopathological characteristics of the liver metastasis** | | | |
| **ER-status (positive vs negative)** | | | |
| **negative** | 7 | 3 (23.1) | 4 (20) |
| **positive** | 26 | 10 (76.9) | 16 (80.0) |
| **Missing** | 1 | 0 | 1 |

| | | | |
|---|---|---|---|
| **HER2-status (amplified vs non-amplified)** | | | |
| **non-amplified** | 29 | 11 (84.6) | 18 (85.7) |
| **amplified** | 5 | 2 (15.4) | 3 (14.3) |
| **Missing** | 1 | 0 | 1 |
| **Extrahepatic metastasis (yes vs no)** | | | |
| **no** | 20 | 9 (90.0) | 11 (73.3) |
| **yes** | 5 | 1 (10.0) | 4 (26.7) |
| **Missing** | 9 | 3 | 6 |
| **Time between BC diagnosis and liver surgery (continuous)** | | | |
| **<1month** | 1 | 0 (0.0) | 1 (6.7) |
| **<1year** | 2 | 2 (22.2) | 0 (0.0) |
| **<2 years** | 2 | 1 (11.1) | 1 (6.7) |
| **≥2years** | 19 | 6 (66.7) | 13 (86.6) |
| **Missing** | 10 | 4 | 6 |

Abbreviations: BC= Breast Cancer; T= primary tumor; N= regional lymph node; M= distant metastasis; c= clinical; p= pathological; CI=Confidence Interval; ER= Estrogen Receptor; HER2 = Human Epidermal Growth Factor Receptor-2; NST= No Special Type; ILC= Invasive Lobular Carcinoma; OR= Odds Ratio.

**References**


1. Salgado R, Denkert C, Demaria S, Sirtaine N, Klauschen F, Pruneri G, et al. The evaluation of tumor-infiltrating lymphocytes (TILs) in breast cancer: recommendations by an International TILs Working Group 2014. Annals of Oncology [Internet]. Oxford University Press; 2015 [cited 2022 Oct 6];26:259. Available from: /pmc/articles/PMC6267863/

2. Latacz E, Höppener D, Bohlok A, Leduc S, Tabariès S, Fernández Moro C, et al. Histopathological growth patterns of liver metastasis: updated consensus guidelines for pattern scoring, perspectives and recent mechanistic insights. British Journal of Cancer 2022 127:6 [Internet]. Nature Publishing Group; 2022 [cited 2022 Oct 5];127:988–1013. Available from: https://www.nature.com/articles/s41416-022-01859-7
