# Supplementary material for: Histopathological growth patterns and tumor-infiltrating lymphocytes in breast cancer liver metastases
Source: NPJ Breast Cancer. 2023 Dec 15;9:100. doi: 10.1038/s41523-023-00602-6 (PMC10724185; doi:10.1038/s41523-023-00602-6)
Supplement: Supplementary file 2 — Reporting Summary [file 41523_2023_602_MOESM2_ESM.pdf]

nature portfolio

Corresponding author(s): Christine Desmedt

Last updated by author(s): Oct 25, 2023

Reporting Summary

Nature Portfolio wishes to improve the reproducibility of the work that we publish. This form provides structure for consistency and transparency in reporting. For further information on Nature Portfolio policies, see our Editorial Policies and the Editorial Policy Checklist.

Statistics

For all statistical analyses, confirm that the following items are present in the figure legend, table legend, main text, or Methods section.

n/a	Confirmed	
☐	☒	The exact sample size (*n*) for each experimental group/condition, given as a discrete number and unit of measurement
☐	☒	A statement on whether measurements were taken from distinct samples or whether the same sample was measured repeatedly
☐	☒	The statistical test(s) used AND whether they are one- or two-sided<br>*Only common tests should be described solely by name; describe more complex techniques in the Methods section.*
☐	☒	A description of all covariates tested
☐	☒	A description of any assumptions or corrections, such as tests of normality and adjustment for multiple comparisons
☐	☒	A full description of the statistical parameters including central tendency (e.g. means) or other basic estimates (e.g. regression coefficient) AND variation (e.g. standard deviation) or associated estimates of uncertainty (e.g. confidence intervals)
☐	☒	For null hypothesis testing, the test statistic (e.g. F, t, r) with confidence intervals, effect sizes, degrees of freedom and P value noted<br>*Give P values as exact values whenever suitable.*
☒	☐	For Bayesian analysis, information on the choice of priors and Markov chain Monte Carlo settings
☒	☐	For hierarchical and complex designs, identification of the appropriate level for tests and full reporting of outcomes
☐	☒	Estimates of effect sizes (e.g. Cohen's *d*, Pearson's *r*), indicating how they were calculated

Our web collection on statistics for biologists contains articles on many of the points above.

Software and code

Policy information about availability of computer code

Data collection	Excel
Data analysis	R version 4.2.2. Packages: 'stringr', 'reshape', 'reshape2', 'ggplot2', 'forestplot', 'dplyr', 'hrbrthemes', 'ggpubr', 'ggsignif', 'finalfit', 'stringr', 'quantreg', 'survcomp', 'DescTools', 'hrbrthemes', 'logistif', 'mcr', 'viridis', 'BlandAltmanLeh'.

For manuscripts utilizing custom algorithms or software that are central to the research but not yet described in published literature, software must be made available to editors and reviewers. We strongly encourage code deposition in a community repository (e.g. GitHub). See the Nature Portfolio guidelines for submitting code & software for further information.

Data

Policy information about availability of data

All manuscripts must include a data availability statement. This statement should provide the following information, where applicable:

- Accession codes, unique identifiers, or web links for publicly available datasets
- A description of any restrictions on data availability
- For clinical datasets or third party data, please ensure that the statement adheres to our policy

The data that support the findings of this study are available upon request to the corresponding author after signature of a Data Access Agreement. Due to the personal nature of the containing information, only the non-sensitive data are publicly available and the sensitive data have been modified before being published on the Code Ocean capsule.

Research involving human participants, their data, or biological material

Policy information about studies with human participants or human data. See also policy information about sex, gender (identity/presentation), and sexual orientation and race, ethnicity and racism.

Reporting on sex and gender	All the patients are female, except two who are male.
Reporting on race, ethnicity, or other socially relevant groupings	No report on race, ethnicity, or other socially relevant groupings.
Population characteristics	We collected samples from 125 unique metastases from 133 patients with BC who underwent surgical resection of their LMs between October 2000 and September 2021, further referred to as the surgical cohort. Secondly, we collected samples from 84 unique LM from 23 BC patients included in two post-mortem tissue donation programs. In the surgical and post-mortem cohorts, the median age at primary diagnosis was 47 (IQR:18) and 50 (IQR:20.75) years, respectively. In both cohorts, most of the primary tumors were invasive breast carcinoma of no special type (IBC-NST) (86%) and expressed ER but not HER2, with 72% (76/106) primary cancers being ER+/HER2 non-amp, 5% (5/106) ER-/HER2 amp, 12% (13/106) ER+/HER2 amp and 11% (12/106) ER-/HER2 non-amp. In 63% (80/127) of the patients, the LM were ER+/HER2 non-amp, in 17% (22/127) ER-/HER2 non-amp, in 14% (18/127) ER+/HER2 amp and in 6% (7/127) ER-/HER2 amp.
Recruitment	The patients who had a diagnosis of BCLM from: 1) a retrospective cohort of 133 patients who underwent surgical resection of their LM in one of the eight participating hospitals (University Hospitals Leuven – Leuven, Belgium; Institut Jules Bordet - Brussels, Belgium; Erasme Hospital - Brussels, Belgium; GZA Ziekenhuizen - Antwerp, Belgium; Centro Hospitalar e Universitario de Coimbra - Coimbra, Portugal; Uzsoki Hospital - Budapest, Hungary; Erasmus MC Cancer Institute - Rotterdam, The Netherlands; Karolinska Institutet - Stockholm, Sweden) between October 2000 and September 2021 (Supplementary Figure 3A), and, 2) a post-mortem cohort of 23 patients from two autopsy programs currently ongoing (UPTIDER, KU/UZ Leuven – Leuven, Belgium, NCT04531696; and Semmelweis University - Budapest, Hungary)
Ethics oversight	This project has been approved by all local Ethics Committees. The study was performed in accordance with the Declaration of Helsinki.

Note that full information on the approval of the study protocol must also be provided in the manuscript.

Field-specific reporting

Please select the one below that is the best fit for your research. If you are not sure, read the appropriate sections before making your selection.

☒ Life sciences ☐ Behavioural & social sciences ☐ Ecological, evolutionary & environmental sciences

For a reference copy of the document with all sections, see nature.com/documents/nr-reporting-summary-flat.pdf

Life sciences study design

All studies must disclose on these points even when the disclosure is negative.

Sample size	The study currently includes clinical data and samples from patients who had a diagnosis of BCLM from: 1) a retrospective cohort of 133 patients who underwent surgical resection of their LM in one of the eight participating hospitals (University Hospitals Leuven – Leuven, Belgium; Institut Jules Bordet - Brussels, Belgium; Erasme Hospital - Brussels, Belgium; GZA Ziekenhuizen - Antwerp, Belgium; Centro Hospitalar e Universitario de Coimbra - Coimbra, Portugal; Uzsoki Hospital - Budapest, Hungary; Erasmus MC Cancer Institute - Rotterdam, The Netherlands; Karolinska Institutet - Stockholm, Sweden) between October 2000 and September 2021 (Supplementary Figure 3A), and, 2) a post-mortem cohort of 23 patients from two autopsy programs currently ongoing (UPTIDER, KU/UZ Leuven – Leuven, Belgium, NCT04531696; and Semmelweis University - Budapest, Hungary)
Data exclusions	Subjects were excluded according to the following criteria:<br>• History of radiofrequency ablation (RF), stereotactic Body Radiotherapy (SBRT) or intra-arterial treatment such as chemoembolization (TACE) or selective internal radiation therapy (SIRT) performed on the metastasis to be examined (accepted if performed in other metastasis);<br>• Had an active autoimmune disease that had required systemic treatment in the past 2 years prior to the surgery (i.e. with use of disease modifying agents, corticosteroids or immunosuppressive drugs). Replacement therapy (e.g., thyroxine, insulin, or physiologic corticosteroid replacement therapy for adrenal or pituitary insufficiency, etc.) is not considered a form of systemic treatment;<br>• Presence of immunodeficiency or immune dysregulatory diseases and conditions which require active immune modulatory treatment of any kind, or required treatment in the past two years from date of diagnosis of breast cancer;<br>• History or current evidence of any condition, therapy, or laboratory abnormality that might confound the results of the trial in the opinion of the treating investigator.
Replication	NA
Randomization	NA
Blinding	NA

Reporting for specific materials, systems and methods

We require information from authors about some types of materials, experimental systems and methods used in many studies. Here, indicate whether each material, system or method listed is relevant to your study. If you are not sure if a list item applies to your research, read the appropriate section before selecting a response.

Materials & experimental systems

n/a	Involved in the study
☐	☐ Antibodies
☐	☐ Eukaryotic cell lines
☐	☐ Palaeontology and archaeology
☐	☐ Animals and other organisms
☐	☒ Clinical data
☐	☐ Dual use research of concern
☐	☐ Plants

Methods

n/a	Involved in the study
☐	☐ ChIP-seq
☐	☐ Flow cytometry
☐	☐ MRI-based neuroimaging

Antibodies

Antibodies used	NA
Validation	NA

Eukaryotic cell lines

Policy information about cell lines and Sex and Gender in Research

Cell line source(s)	NA
Authentication	NA
Mycoplasma contamination	NA
Commonly misidentified lines (See ICLAC register)	NA

Palaeontology and Archaeology

Specimen provenance	NA
Specimen deposition	NA
Dating methods	NA

☐ Tick this box to confirm that the raw and calibrated dates are available in the paper or in Supplementary Information.

Ethics oversight	NA

Note that full information on the approval of the study protocol must also be provided in the manuscript.

Animals and other research organisms

Policy information about studies involving animals; ARRIVE guidelines recommended for reporting animal research, and Sex and Gender in Research

Laboratory animals	NA
Wild animals	NA
Reporting on sex	NA
Field-collected samples	NA

Ethics oversight | NA

Note that full information on the approval of the study protocol must also be provided in the manuscript.

Clinical data

Policy information about clinical studies

All manuscripts should comply with the ICMJE guidelines for publication of clinical research and a completed CONSORT checklist must be included with all submissions.

Clinical trial registration: This project has been approved by all local Ethics Committees. The study was performed in accordance with the Declaration of Helsinki. UPTIDER project received the approval of the local Ethics Committee (S64813;28/08/2022) from UZ Leuven, Belgium.

Study protocol: The data that support the findings of this study are available upon request to the corresponding author after signature of a Data Access Agreement. The data are not publicly available due to the personal nature of the containing information.

Data collection: The study currently includes clinical data and samples from patients who had a diagnosis of BCLM from: 1) a retrospective cohort of 133 patients who underwent surgical resection of their LM in one of the eight participating hospitals (University Hospitals Leuven – Leuven, Belgium; Institut Jules Bordet - Brussels, Belgium; Erasme Hospital - Brussels, Belgium; GZA Ziekenhuizen - Antwerp, Belgium; Centro Hospitalar e Universitario de Coimbra - Coimbra, Portugal; Uzsoki Hospital - Budapest, Hungary; Erasmus MC Cancer Institute - Rotterdam, The Netherlands; Karolinska Institutet - Stockholm, Sweden) between October 2000 and September 2021 (Supplementary Figure 3A), and, 2) a post-mortem cohort of 23 patients from two autopsy programs currently ongoing (UPTIDER, KU/UZ Leuven – Leuven, Belgium, NCT04531696; and Semmelweis University - Budapest, Hungary)

Outcomes: The associations between HGP and progression-free (PFS) and overall survival (OS) were visualized using Kaplan-Meier curves and further assessed using univariable and multivariable Cox proportional hazard regressions. PFS was defined as the time from first liver resection to the time of progression or death, and OS was defined as the time from first liver resection to the time of death of any cause.

Dual use research of concern

Policy information about dual use research of concern

Hazards

Could the accidental, deliberate or reckless misuse of agents or technologies generated in the work, or the application of information presented in the manuscript, pose a threat to:

No	Yes	
☐	☒	Public health
☒	☐	National security
☒	☐	Crops and/or livestock
☒	☐	Ecosystems
☒	☐	Any other significant area

Hazards: NA

For examples of agents subject to oversight, see the United States Government Policy for Institutional Oversight of Life Sciences Dual Use Research of Concern.

Experiments of concern

Does the work involve any of these experiments of concern:

No	Yes	
☒	☐	Demonstrate how to render a vaccine ineffective
☒	☐	Confer resistance to therapeutically useful antibiotics or antiviral agents
☒	☐	Enhance the virulence of a pathogen or render a nonpathogen virulent
☒	☐	Increase transmissibility of a pathogen
☒	☐	Alter the host range of a pathogen
☒	☐	Enable evasion of diagnostic/detection modalities
☒	☐	Enable the weaponization of a biological agent or toxin
☒	☐	Any other potentially harmful combination of experiments and agents

Precautions and benefits

Biosecurity precautions	NA
Biosecurity oversight	NA
Benefits	NA
Communication benefits	NA

Plants

Seed stocks	NA
Novel plant genotypes	NA
Authentication	NA

ChIP-seq

Data deposition

- [ ] Confirm that both raw and final processed data have been deposited in a public database such as GEO.
- [ ] Confirm that you have deposited or provided access to graph files (e.g. BED files) for the called peaks.

Data access links<br>*May remain private before publication.*	NA
Files in database submission	NA
Genome browser session<br>(e.g. UCSC)	NA

Methodology

Replicates	NA
Sequencing depth	NA
Antibodies	NA
Peak calling parameters	NA
Data quality	NA
Software	NA

Flow Cytometry

Plots

Confirm that:

- [ ] The axis labels state the marker and fluorochrome used (e.g. CD4-FITC).
- [ ] The axis scales are clearly visible. Include numbers along axes only for bottom left plot of group (a 'group' is an analysis of identical markers).
- [ ] All plots are contour plots with outliers or pseudocolor plots.
- [ ] A numerical value for number of cells or percentage (with statistics) is provided.

Methodology

Sample preparation	NA

Instrument	NA
Software	NA
Cell population abundance	NA
Gating strategy	NA

☐ Tick this box to confirm that a figure exemplifying the gating strategy is provided in the Supplementary Information.

Magnetic resonance imaging

Experimental design

Design type	NA
Design specifications	NA
Behavioral performance measures	NA

Acquisition

Imaging type(s)	NA
Field strength	NA
Sequence & imaging parameters	NA
Area of acquisition	NA

Diffusion MRI ☐ Used ☒ Not used

Preprocessing

Preprocessing software	NA
Normalization	NA
Normalization template	NA
Noise and artifact removal	NA
Volume censoring	NA

Statistical modeling & inference

Model type and settings	NA
Effect(s) tested	NA

Specify type of analysis: ☐ Whole brain ☐ ROI-based ☐ Both

Statistic type for inference (See Eklund et al. 2016)	NA
Correction	NA

Models & analysis

n/a	Involved in the study
☒	☐ Functional and/or effective connectivity
☒	☐ Graph analysis
☒	☐ Multivariate modeling or predictive analysis
